# Methadone maintenance treatment and mortality in people with criminal convictions: A population-based retrospective cohort study from Canada

**DOI:** 10.1371/journal.pmed.1002625

**Published:** 2018-07-31

**Authors:** Angela Russolillo, Akm Moniruzzaman, Julian M. Somers

**Affiliations:** Somers Research Group, Faculty of Health Sciences, Simon Fraser University, Burnaby, British Columbia, Canada; University of Queensland, AUSTRALIA

## Abstract

**Background:**

Individuals with criminal histories have high rates of opioid dependence and mortality. Excess mortality is largely attributable to overdose deaths. Methadone maintenance treatment (MMT) is one of the best evidence-based opioid substitution treatments (OSTs), but there is uncertainty about whether methadone treatment reduces the risk of mortality among convicted offenders over extended follow-up periods. The objective of this study was to investigate the association between adherence to MMT and overdose fatality as well as other causes of mortality.

**Methods and findings:**

We conducted a retrospective cohort study involving linked population-level administrative data among individuals in British Columbia (BC), Canada with a history of conviction and who filled a methadone prescription between January 1, 1998 and March 31, 2015. Participants were followed from the date of first-dispensed methadone prescription until censoring (date of death or March 31, 2015). Methadone was divided into medicated (methadone was dispensed) and nonmedicated (methadone was not dispensed) periods and analysed as a time-varying exposure. Hazard ratios (HRs) with 95% CIs were estimated using multivariable Cox regression to examine mortality during the study period. All-cause and cause-specific mortality rates were compared during medicated and nonmedicated methadone periods. Participants (*n* = 14,530) had a mean age of 34.5 years, were 71.4% male, and had a median follow-up of 6.9 years. A total of 1,275 participants died during the observation period. The overall all-cause mortality rate was 11.2 per 1,000 person-years (PYs). Participants were significantly less likely to die from both nonexternal (adjusted HR [AHR] 0.27 [95% CI 0.23–0.33]) and external (AHR 0.41 [95% CI 0.33–0.51]) causes during medicated periods, independent of sociodemographic, criminological, and health-related factors. Death due to infectious diseases was 5 times lower (AHR 0.20 [95% CI 0.13–0.30]), and accidental poisoning (overdose) deaths were nearly 3 times lower (AHR 0.39 [95% CI 0.30–0.50]) during medicated periods. A competing risk regression demonstrated a similar pattern of results. The use of a Canadian offender population may limit generalizability of results. Furthermore, our observation period represents community-based methadone prescribing and may omit prescriptions administered during hospital separations. Therefore, the magnitude of the protective effects of methadone from nonexternal causes of death should be interpreted with caution.

**Conclusions:**

Adherence to methadone was associated with significantly lower rates of death in a population-level cohort of Canadian convicted offenders. Achieving higher rates of adherence may reduce overdose deaths and other causes of mortality among offenders and similarly marginalized populations. Our findings warrant examination in other study centres in response to the crisis of opiate-involved deaths.

## Abstract 2

In this retrospective study, Angela Russolillo and colleagues investigate mortality rates among people with a drug addiction and a criminal record during periods of methadone treatment and periods without drug substitution therapy.

## Author summary

**Why was this study done?:**

- Individuals with criminal histories experience high rates of opioid dependence and premature mortality.
- Deaths caused by opioids are rising acutely and offenders are at risk.
- Adherence to opioid substitution treatments (OSTs), such as methadone, has been shown to reduce the risk of death during custody and immediately following release.
- Little is known about the long-term association between methadone adherence and mortality.

**What did researchers do and find?:**

- This study integrated population-level data including prescriptions, convictions, and deaths in British Columbia (BC), Canada spanning 1998–2015.
- We examined the risk of all-cause and cause-specific death among 14,530 people with criminal convictions who had been prescribed methadone.
- Overall and cause-specific mortality rates were compared between periods when methadone was and was not dispensed.
- Periods when methadone was dispensed were associated with lower risk of mortality, including overdose fatalities, after controlling for covariates.

**What do these findings mean?:**

- Practices to increase methadone adherence among opioid-dependent offenders are required and may reduce overdose-related, as well as other causes of, premature death.

## Introduction

Overdoses and deaths caused by opioids have been declared a public health emergency in North America. The rising prevalence of opioid dependence [1], alongside the emergence of fentanyl in the illicit drug market [2], is contributing to premature mortality and sparking an urgent need to mobilize public health and public safety resources. Many of North America’s leading health organisations (American Medical Association, Health Canada, and Centers for Disease Control and Prevention) have set priorities in response to the escalating public health crisis [3]. Interventions emphasize prevention, education, and comprehensive care, including access to substitution treatment where indicated [4]. Particular attention has been directed toward high-risk populations, including offenders. Accidental poisoning is the most common cause of mortality among opioid-dependent individuals [5,6], with opioids present in the vast majority of drug-related deaths among ex-prisoners [7]. Several mortality-related risk factors are overrepresented among offenders (e.g., repeated incarceration, low socioeconomic status, and homelessness) [8,9], compounding the hazards associated with substance misuse. The prevalence of opioid dependence [10] and risk of death from illicit drugs [11,12], such as heroin, is higher among offenders and is acutely elevated in the weeks following prison release [13,14]. Despite evidence that prevention and treatment options (e.g., methadone) may reduce the risk of death among opioid-dependent individuals [15,16], there remain significant barriers [17,18] and underutilisation [19] of substitution treatment options for offenders. Factors such as stigma, insufficient pharmacotherapy knowledge, concerns related to medication diversion, and poor links between corrections and community-based care providers can restrict access to methadone maintenance treatment (MMT) and continuity of care for offenders with opioid dependence [20,21] whether they are sentenced to custody or community settings, as well as following the completion of sentencing.

MMT remains one of the best researched and most widely used opioid substitution treatments (OSTs) [22,23]. MMT engagement is associated with reduced illicit opioid use [24], infectious disease transmission [25], and recidivism [26,27]. While the benefits of MMT adherence are well established for a number of health and justice outcomes, including reduced health care costs [28], the role of MMT adherence in mortality among offenders is less clear. A number of observational studies in Europe and Australia have indicated that adherence to methadone reduces the risk of death during treatment compared with periods of nontreatment [29–33] in general opiate-dependent populations. In these studies, treatment effects of methadone are strongest for drug-related deaths [31] and among subpopulations of MMT users with infectious diseases (e.g., by potentiating adherence to antiretroviral treatments [ARTs]) [34]. However, these studies are not specific to offenders (in or out of custody) and are drawn from relatively small samples sizes [30,31,35], with maximum follow-up periods of 4 to 7 years [29,33,36]. Among studies that do focus on MMT among offenders, most concentrate on mortality in the initial postrelease period [16,37] or during custody [38,39]. However, in many jurisdictions, including British Columbia (BC), the majority of convictions result in sentences served exclusively in the community (versus prison) and are of relatively short duration. Little is known about the long-term course and impact of MMT among people who have served sentences at some point in their lives, although available evidence confirms that MMT adherence fluctuates over time [40]. Despite clinical and empirical support from observational research supporting a broad range of protective effects, independent systematic reviews evaluating the association between methadone treatment and mortality have concluded that the available evidence is “weak” [41] and “suggestive” [24]. Moreover, the very limited body of experimental evidence is equivocal. Mattick and colleagues [22] reported positive but nonsignificant associations between mortality and methadone compared with nontreatment. In contrast, a recent meta-analysis [32] reported greater reductions in mortality with methadone compared with buprenorphine.

Careful examination of MMT and mortality among offenders is particularly valuable due to elevated risks in this population (e.g., injection drug use, HIV) [42], periods of potential interruptions in treatment (e.g., incarceration) [43], barriers to treatment [44], and high likelihood of relapse [45]. In this study, we investigated the association between methadone and mortality in the population of convicted offenders in BC, Canada over a 17-year observation period. We describe the distribution of nonexternal and external causes of morbidity and mortality and address 2 main questions related to MMT: is the risk of all-cause mortality lower during periods of dispensed methadone compared with nondispensed periods? Is the risk of overdose mortality lower during periods of dispensed methadone?

## Methods

### Study design and population

Data were obtained by linking population-level administrative records in BC, Canada under the Inter-Ministry Research Initiative (IMRI). The study cohort consisted of all individuals with provincial justice contacts (*n* = 250,884) in BC. Individuals with a history of conviction and who filled a methadone prescription between January 1, 1998 and March 31, 2015 were eligible for inclusion. Citizens of BC are legally required to register with the province’s publicly funded health system and are assigned a unique ID. This ID is used to link information from different program areas. We used several comprehensive data sets: the Ministry of Health’s PharmaNet, Vital Statistics, and Billing databases; and the Ministry of Justice’s registry of convictions. The IMRI serves as a resource for the development of policies and services that span health, justice, and social welfare sectors. Details of the IMRI that are not essential to the current study have been described elsewhere [46].

**Fig 1:**
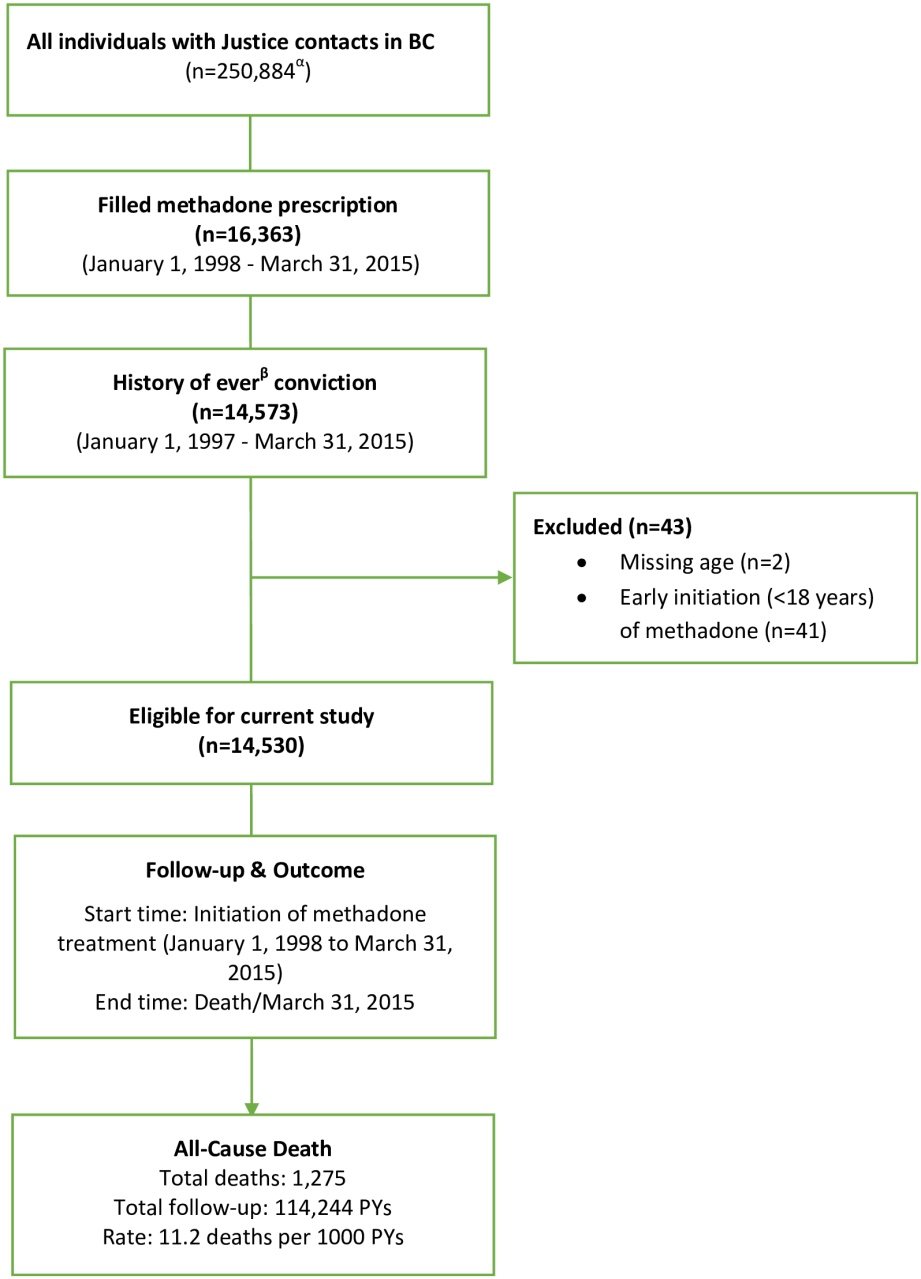
Flow chart of offenders included in the study. ^α^The cohort included participants (offenders) who had convictions (found or plead guilty and sentenced) as well as those (nonoffenders) who did not have any convictions but were under supervision of the Ministry of Justice due to remand or bail and later found not guilty. ^β^This time period included the study/exposure period (January 1, 1998 to March 31, 2015) for methadone as well as time prior to enrolment (from the time when justice databases became available, January 1997). BC, British Columbia; PY, person-year.

Follow-up extended from the date of first-dispensed methadone prescription until censoring (date of death or March 31, 2015) (Fig 1). Methadone prescription transactions were collected by the Ministry of Health. Corrections-related incidents and sociodemographic variables (age, gender, ethnicity, and education) were collected by the Ministry of Justice. Mortality data were obtained from the BC Vital Statistics Agency. Covariate information concerning medical and lab service use was extracted from the Provincial Medical Services Plan database, which details the date, diagnostic code, and cost associated with medical services to citizens in BC, including while serving sentences under provincial corrections. The study was approved by the Simon Fraser University Research Ethics Board.

### Variables

Data on the main exposure, methadone, were extracted from the PharmaNet database, a province-wide network linking all prescriptions issued by BC pharmacies. This register omits dispensing information during hospitalisation or outside the province of BC. Authorized physicians who hold an exemption from Health Canada are permitted to prescribe methadone in BC [47]. Methadone is dispensed to individuals who meet criteria for opioid dependence as defined by the Diagnostic and Statistical Manual of Mental Disorders (DSM), 5th edition and/or DSM-IV-TR. Patients receiving methadone are required to comply with daily witnessed ingestion under the supervision of a pharmacist (i.e., attend pharmacy daily to receive dispensed dose of methadone), unless authorized to hold ‘carry’ privileges [47].

Methadone was treated as a time-varying exposure (i.e., medication status was not constant throughout follow-up), and each participant’s follow-up was divided into medicated (methadone was dispensed) and nonmedicated (methadone was not dispensed) periods. Following the method used in previous research [27], a participant was considered exposed to methadone based on pharmacy fill transaction dates (see S1 Text). If a participant filled their methadone prescription consistently (no gap in pharmacy transaction dates) for a period of time, this was treated as a single interval and considered a medicated period (methadone was dispensed). If a participant didn’t fill a prescription for a period of time (gap in pharmacy transaction dates), the interval was considered a nonmedicated period (methadone was not dispensed). Participants were expected to alternate between medicated and nonmedicated periods (for further details see S1 Text).

The main outcome was death during follow-up. When a person dies in BC, medical personnel (physician or nurse practitioner) or a coroner must complete a death certificate, and the death must be registered with the Vital Statistics Agency. Causes of death were coded according to the International Statistical Classification of Diseases and Related Health Problems 10th Revision (ICD-10). We extracted details of all-cause mortality and cause-specific mortality separated by ICD-10 chapter in accordance with the recorded cause of death. Within the category of nonexternal causes of death (ICD-10 chapter I to XVII), we separately examined deaths from infectious disease, and within external causes of death (ICD-10 chapter XX), we examined deaths by accidental poisoning and intentional self-harm (i.e., suicide).

Several covariates were included, including age (at time of methadone initiation), gender, ethnicity, education, initiation of methadone period (years), prior offences (year preceding methadone initiation), number of offences after methadone initiation (time-varying), number of custody admissions (available from 2007–2015 and used for subgroup analysis only) after methadone initiation (time-varying), severe mental illness, prior service use for non–substance-related mental disorders, prior service use for substance use disorders, and prior service use for nonpsychiatric medical reasons (see S1 Text for details).

### Statistical analyses

We used descriptive statistics (counts and proportions for nominal variables; mean and SD, or median and interquartile range [IQR], for continuous variables) to characterize the study sample. We chose time-to-event, or survival, analysis because our outcome of interest was not only the occurrence of an event (death) but also when the event occurred [48]. Methadone was our primary covariate and was time-varying during the follow-up period. To address this time-varying effect, we used the extended Cox model [49]. As an estimate of effect size, we reported the hazard ratios (HRs) along with 95% CIs.

To control for potential confounding, HRs were estimated using multivariable Cox regression, with adjustment for age, gender, ethnicity, education, methadone initiation period, psychiatric diagnoses, criminal history, and health service use. In the Cox regression, we assessed the proportional hazards assumption using Kaplan Meier curve as well as the Schoenfeld residuals [50,51]. We found no violation of proportionality for methadone in the Cox models. We used the robust variance estimator to estimate SEs for the parameters [52,53]. We chose the conventional alpha level (*p* ≤ 0.05) to report significance for the estimated parameters. All reported *p*-values were 2-sided.

We used the Cox model for cause-specific deaths and also conducted competing risk regression analysis [54–56] as a sensitivity analysis. We performed competing risk regression using the method proposed by Fine and Gray [57]. Additional sensitivity analyses were conducted inflating the definition of last-dispensed methadone prescription from 1 day to 3 and 7 days and among participants whose cause of death was HIV. A subgroup analysis was conducted among participants who initiated methadone and had at least one custody admission restricted to the years 2007 to 2015, when admission and release dates were deemed to be sufficiently reliable.

Individuals with missing demographic information, including ethnicity and education level, were not excluded from the analysis but were included as separate categories titled ‘unknown’ ethnicity and ‘unknown’ education level. STATA 13.1 was used to conduct all analyses.

## Results

The study cohort included 14,530 convicted offenders (mean [SD] age 34.5 [9.4] years; 71.4% male) followed from January 1, 1998 to March 31, 2015 for a total of 114,243.7 person-years (PYs). Table 1 shows baseline sociodemographic and criminological information as well as diagnostic and medical services details for the eligible sample. For methadone prescriptions, the median number of medicated and nonmedicated periods in years were 2.0 (IQR 0.5–4.9) and 3.2 (IQR 0.9–7.1), respectively, representing a total medicated time of 47,681.7 PYs and a nonmedicated time of 66,562.0 PYs.

**Table 1:** Sociodemographic, methadone, and crime-related characteristics of 14,530 convicted offenders from BC, 1998–2015.

Variable	Mean (SD)/*n* (%)
***Age at enrolment***^1^	
Mean (SD)	34.5 (9.4)
Median (IQR)	33.3 (27.0–41.0)
Min, Max	18.0, 74.9
***Age groups (years)***	
18 < 25	2,484 (17.1)
25 < 35	5,633 (38.8)
35 < 45	4,242 (29.2)
45 < 55	1,849 (12.7)
≥55	322 (2.2)
***Men*, *n (%)***	10,378 (71.4)
***Ethnicity*, *n (%)***	
White	10,546 (72.6)
Indigenous	2,180 (15.0)
Other	1,300 (8.9)
Unknown	504 (3.5)
***Education level*, *n (%)***	
<Grade 10	1,930 (13.3)
Grade 10/11	5,028 (34.6)
Grade 12	4,869 (33.5)
Vocational/university	1,668 (11.5)
Unknown	1,035 (7.1)
***Follow-up period*, *in years***	
Mean (SD)	7.9 (5.1)
Median (IQR)	6.9 (3.4–12.8)
Min, Max	<0.1, 17.2
Total follow-up time (PYs)	114, 243.7
***Year of methadone initiation*, *n (%)***	
1998 to 2000	2,844 (19.6)
2001 to 2005	3,311 (22.8)
2006 to 2010	4,313 (29.7)
2011 to 2015^2^	4,062 (27.9)
***Medicated period*, *in years***	
Mean (SD)	3.3 (3.6)
Median (IQR)	2.0 (0.5–4.9)
Min, Max	<0.1, 16.9
Total medicated time, in PYs	47, 681.7
***Number of medicated periods/episodes***	
Mean (SD)	44.4 (58.6)
Median (IQR)	23 (7–59)
Min, Max	1, 638
***Nonmedicated period*, *in years***	
Mean (SD)	4.6 (4.4)
Median (IQR)	3.2 (0.9–7.1)
Min, Max	0.0, 17.2
Total nonmedicated time, in PYs	66, 562.0
***Number of nonmedicated periods/episodes***^3^	
Mean (SD)	44.0 (58.5)
Median (IQR)	23 (7–58)
Min, Max	0, 638
***Number of methadone* transactions *in the year after enrolment (n = 13*,*490)***^4^**, *mean (SD)***	160.8 (116.4)
***Received buprenorphine or buprenorphine-naloxone in follow-up period*, *n (%)***	1,096^5^ (7.5)
***Pharmacy transactions in the year after enrolment (n = 1*.*055)***^6^**, *mean (SD)***	
Number of buprenorphine or buprenorphine-naloxone transactions	6.9 (28.2)
Number of methadone transactions	149.8 (109.5)
***Severe mental illness***	
No schizophrenia or bipolar	9,548 (65.7)
Schizophrenia	2,217 (15.3)
Bipolar	2,765 (19.0)
***Number of offences in the year prior to enrolment*, *mean (SD)***	1.1 (2.3)
***Any offence in the year prior to enrolment*, *n (%)***	
None	9032 (62.2)
1–2 offences	3,373 (23.2)
>2 offences	2,125 (14.6)
***Any jail sentence in the year prior to enrolment*, *n (%)***	2,824 (19.4)
***MSP services (NSMD related) in the 5-year period prior to enrolment*, *n (%)***	
Low^7^ (≤2)	7,388 (50.9)
Medium (3–10)	3,745 (25.8)
High (≥11	3,397 (23.3)
***MSP services (SUD related) in the 5-year period prior to enrolment*, *n (%)***	
Low^8^ (≤4)	7,539 (51.9)
Medium (5–13)	3,427 (23.6)
High (≥14)	3,564 (24.5)
***MSP services (nonpsychiatric) in the 5-year period prior to enrolment*, *n (%)***	
Low^9^ (≤69)	7,132 (50.3)
Medium (70–139)	3,599 (24.8)
High (≥140)	3,619 (24.9)

^1^Age at enrolment was based on date of initiation of methadone (between January 1, 1998 and March 31, 2015).

^2^2015 included only 3 months (January to March) of data.

^3^A total of 156 (1.1%) participants did not have any nonmedicated periods and received methadone during the entire observation period.

^4^Restricted to participants (*n* = 13,490) who had at least 1 year of follow-up.

^5^Only a single participant received buprenorphine, and the rest received buprenorphine-naloxone.

^6^Restricted to participants (*n* = 1,055) who received buprenorphine or buprenorphine-naloxone and had at least 1 year of follow-up.

^7^50th and 75th percentile were used to categorize into low, medium, and high groups.

^8^50th and 75th percentile were used to categorize into low, medium, and high groups.

^9^50th and 75th percentile were used to categorize into low, medium, and high groups.

Abbreviations: BC, British Columbia; IQR, interquartile range; Max, maximum; Min, minimum; MSP, Medical Services Plan; NSMD, Non–substance-related mental disorder; PY, person-year; SUD, substance use disorder.

During a median follow-up time of 6.9 years (IQR 3.4–12.8), 1,275 participants died (see Table 2). Median age at death was 45.1 (minimum, maximum: 21.3, 75.2). The overall all-cause mortality rate was 11.2 per 1,000 PYs, and the rate was higher during nonmedicated periods (15.0 per 1,000 PYs) compared with medicated periods (5.9 per 1,000 PYs). A total of 504 deaths (39.5%) were attributed to external causes or morbidity and mortality, which were predominantly accidental poisoning (27.8%) and intentional self-harm (4.2%). Infectious diseases (14.9%) and cancer (11.2%) were other major causes of death classified as nonexternal causes. Descriptive statistics characterizing events in the time leading up to death (*n* = 1,275) are available in supporting information (see S1 Table).

**Table 2:** Age^1^ at death according to ICD-10 cause of mortality among 1,275 convicted offenders from BC, 1998–2015.

Cause of Death	*N* (%)	ICD-10 code^2^	Mean (SD)	Median (Min, Max)
***Nonexternal causes of morbidity and mortality (Chap I to XVIII)***^3^				
Certain infectious and parasitic diseases (Chap I)	190 (14.9)	A00-B99	44.8 (9.4)	51.7 (25,1, 67.2)
Neoplasms (Chap II)	143 (11.2)	C00-D48	54.2 (8.1)	53.8 (26.6, 74.9)
Endocrine, nutritional, and metabolic diseases (Chap IV)	15 (1.2)	E00-E90	50.9 (8.6)	51.0 (40.0, 66.5)
Mental and behavioural disorders (Chap V)	50 (3.9)	F00-F99	44.1 (10.6)	43.8 (21.4, 72.7)
Diseases of the nervous system (Chap VI)	17 (1.3)	G00-G99	41.5 (11.0)	41.9 (21.6, 64.7)
Diseases of the circulatory system (Chap IX)	111 (8.7)	I00-I99	47.5 (12.1)	46.7 (22.7, 75.2)
Diseases of the respiratory system (Chap X)	81 (6.3)	J00-J99	51.3 (10.3)	52.9 (22.4, 71.8)
Diseases of the digestive system (Chap XI)	52 (4.1)	K00-K93	53.2 (7.9)	54.9 (30.2, 70.3)
Symptoms, signs, and abnormal clinical and laboratory findings not elsewhere classified (Chap XVIII)	95 (7.5)	R00-R99	41.3 (9.6)	41.0 (21.6, 61.3)
Other nonexternal causes^4^	17 (1.3)	Chap III: D50-D89; Chap XII: L00-L99; Chap XIII: M00-M99; Chap XIV: N00-N99; Chap XVII: Q00-Q99	44.4 (12.6)	44.8 (27.2, 71.8)
***External causes of morbidity and mortality (Chap XX)***^5^		V01-Y98		
Transport accidents	31 (2.4)	V01 to V99	41.6 (11.5)	41.7 (21.5, 65.8)
Falls/accidental drowning/fire	11 (1.0)	W00-W19; W65-W74; X00-X09	46.6 (9.9)	47.0 (34.1, 63.3)
Accidental poisoning	355 (27.8)	X40 to X49	41.7 (9.7)	41.3 (22.2, 67.6)
Intentional self-harm	53 (4.2)	X60 to X84	40.4 (10.0)	41.1 (22.4, 65.8)
Assault	28 (2.2)	X85-Y09	34.6 (6.7)	35.0 (22.9, 43.9)
All other external causes	26 (2.0)	W20-W64; W75-W99; X10-X39; X50-X59; Y10-Y89	42.3 (10.7)	42.5 (21.3, 59.5)
**Total**	1,275 (100)		45.2 (10.9)	45.1 (21.3, 75.2)

^1^Age at the time of death.

^2^lCD-10 codes were used to classify 1,268 deaths, and ICD-9 codes were used to classify 7 deaths, whose comparable ICD-10 group was as follows: Chap 5: 1; Chap X: 1; Chap XVIII: 1; and Chap XX, Accidental poisoning: 4.

^3^This group represents 771 (60.5%) deaths (ICD-10: 768 and ICD-9: 3).

^4^Deaths included: Chap III: 2; Chap VII: 0; Chap VIII: 0; Chap XII: 4; Chap XIII: 5; Chap XIV: 5; Chap XV: 0; Chap XVI: 0; and Chap XVII: 1.

^5^This group represents 504 (39.5%) deaths (ICD-10: 500 and ICD-9: 4).

Abbreviations: BC, British Columbia; ICD-10, International Statistical Classification of Diseases and Related Health Problems 10th Revision.

**Fig 2:**
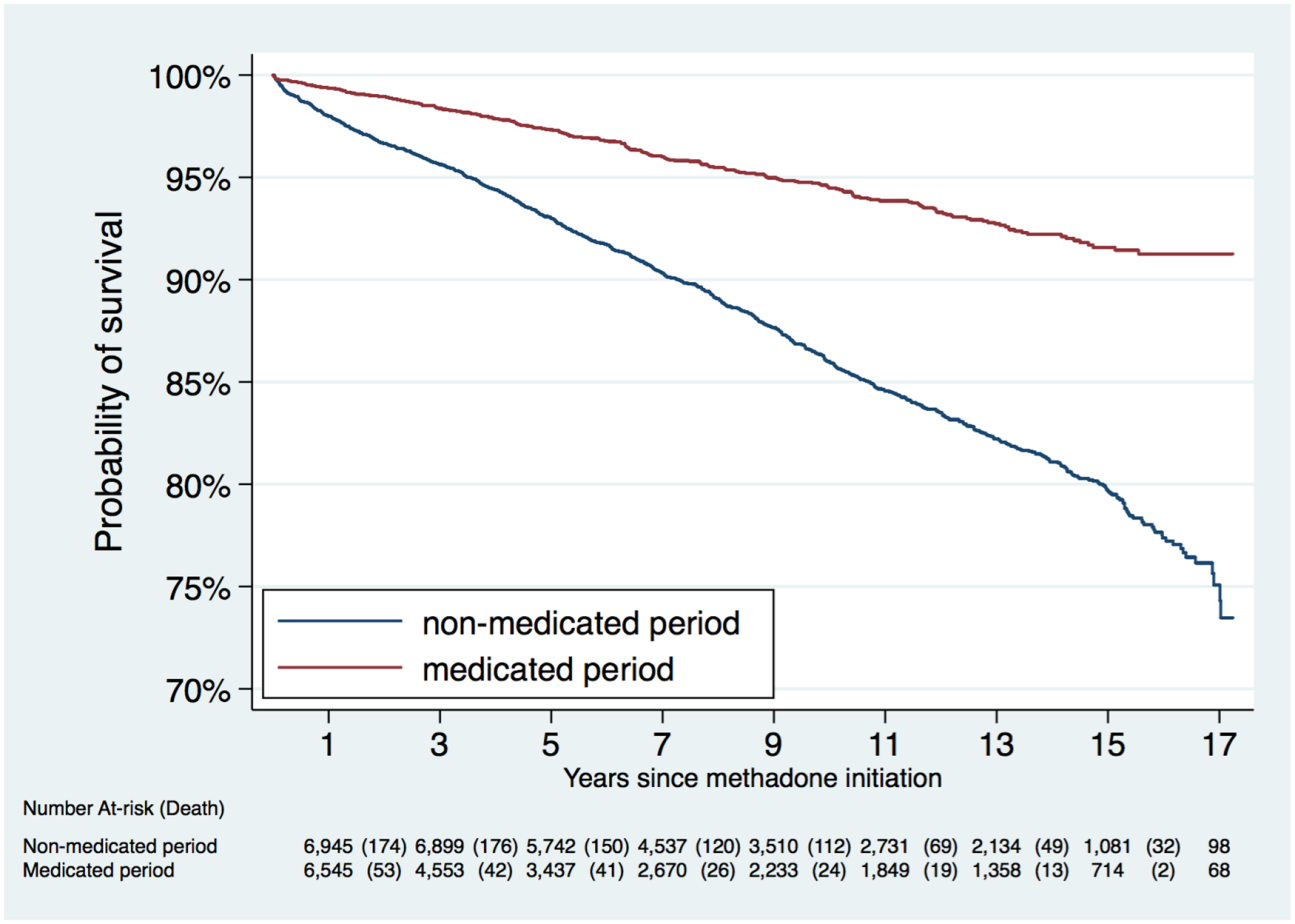
Kaplan-Meier curve for all-cause mortality among 14,530 convicted offenders from BC, 1998–2015. BC, British Columbia.

We compared rates of death during dispensed methadone periods and nondispensed periods. Fig 2 shows the Kaplan-Meier curve for all-cause mortality throughout the follow-up period. Participants were significantly more likely to die during nonmedicated methadone periods than during medicated periods (Fig 2).

As shown in Table 3, participants were more likely to die from both nonexternal (adjusted hazard ratio [AHR] 0.27 [0.23–0.33]) and external (AHR 0.41 [0.33–0.51]) causes during nonmedicated periods. The risk of death due to infectious diseases was 5 times lower (AHR 0.20 [0.13–0.30]) during medicated methadone periods compared with nonmedicated periods. Similarly, for deaths caused by accidental poisoning and intentional self-harm, the AHRs were 0.39 (0.30–0.50) and 0.36 (0.18–0.70), respectively, representing a roughly 2.75 times lower risk of death during medicated methadone periods. All other external (AHR 0.54 [0.34–0.85]) and nonexternal (AHR 0.30 [0.25–0.37]) causes of morbidity and mortality were associated with significantly lower mortality risk during periods when methadone was dispensed. The effects (AHR) of all other covariates included in the multivariable model are available in Supporting information (S2–S4 Tables).

**Table 3:** HR estimates of dispensed methadone on mortality among 14,530 convicted offenders from BC, 1998–2015.

Cause of Death	Medicated Methadone Period	Number of Deaths	Total PYs	Death Rate per 1,000 PYs (95% CI)	UHR (95% CI)^1^	AHR^2^ (95% CI)
All-cause mortality^3^	No	996	66,562.0	15.0 (14.1–15.9)	Reference	Reference
	Yes	279	47,681.7	5.9 (5.2–6.6)	**0.37 (0.32–0.42)**^5^	**0.32 (0.28–0.37)**
	Total^4^	1,275	114,243.7	11.2 (10.6–11.8)		
***Nonexternal causes***	No	623	66,562.0	9.4 (8.7–10.1)	Reference	Reference
	Yes	148	47,681.7	3.1 (2.6–3.7)	**0.32 (0.26–0.38)**	**0.27 (0.23–0.33)**
	Total	771	114,243.7	6.8 (6.3–7.2)		
Infectious diseases	No	162	66,562.0	2.4 (2.1–2.8)	Reference	Reference
	Yes	28	47,681.7	0.6 (0.4–0.9)	**0.23 (0.15–0.35)**	**0.20 (0.13–0.30)**
	Total	190	114,243.7	1.7 (1.4–1.9)		
Other nonexternal causes	No	461	66,562.0	6.9 (6.3–7.6)	Reference	Reference
	Yes	120	47,681.7	2.5 (2.1–3.0)	**0.35 (0.28–0.43)**	**0.30 (0.25–0.37)**
	Total	581	114,243.7	5.1 (4.7–5.5)		
***External causes***	No	373	66,562.0	5.6 (5.1–6.2)	Reference	Reference
	Yes	131	47,681.7	2.8 (2.3–3.3)	**0.45 (0.37–0.55)**	**0.41 (0.33–0.51)**
	Total	504	114,243.7	4.4 (4.0–4.8)		
Accidental poisoning	No	266	66,562.0	4.0 (3.5–4.5)	Reference	Reference
	Yes	89	47,681.7	2.0 (1.9–2.3)	**0.43 (0.33–0.55)**	**0.39 (0.30–0.50)**
	Total	355	114,243.7	3.1 (2.8–3.5)		
Intentional self-harm	No	41	66,562.0	0.6 (0.5–0.8	Reference	Reference
	Yes	12	47,681.7	0.3 (0.1–0.4)	**0.40 (0.21–0.77)**	**0.36 (0.18–0.70)**
	Total	53	114,243.7	0.5 (0.4–0.6)		
Other external causes	No	66	66,562.0	1.0 (0.8–1.3)	Reference	Reference
	Yes	30	47,681.7	0.6 (0.4–0.9)	**0.57 (0.37–0.90)**	**0.54 (0.34–0.85)**
	Total	96	114,243.7	0.8 (0.7–1.03)		

^1^Robust estimator was used to calculate SE and the CIs for both UHR and AHR estimates.

^2^Separate multivariable Cox regression was conducted for all-cause and for each cause-specific death. Each multivariable model was controlled for the following: age (18 < 25 years, 25 < 35 years, 35 < 45 years, 45 < 55 years, and ≥55), gender (men and women), ethnicity (white, indigenous, and other), education (<grade 10, grade 10/11, grade 12, vocational/university), initiation period (1998 to 2000, 2001 to 2005, 2006 to 2010, and 2011 to 2015), prior offences (none, 1–2 offences, and >2 offences), number of current offences (continuous), severe mental illness (no schizophrenia or bipolar; schizophrenia and bipolar), prior NSMD-related services (low, medium, and high), prior SUD-related services (low, medium, and high), and prior nonpsychiatric services (low, medium, and high).

^3^Nonexternal and external causes represent 2 broad subcategories of all-cause mortality (771+ 504 = 1,275). Nonexternal and external causes are further subdivided into 2 (infectious diseases and other nonexternal causes) and 3 (accidental poisoning, intentional self-harm, and other external causes) groups, respectively.

^4^The total represents the sum of deaths for medicated (methadone dispensed) and nonmedicated (methadone not dispensed) periods.

^5^Bold indicated significance of HR estimates at *p* < 0.05.

Abbreviations: AHR, adjusted HR; BC, British Columbia; HR, hazard ratio; NSMD, non–substance-related mental disorder; PY, person-year; SUD, substance use disorder; UHR, unadjusted HR.

A competing risk regression demonstrated a similar pattern of results for both nonexternal (AHR 0.32 [0.27–0.39]) and external causes (AHR 0.46, [0.38–0.57]) of death as well as for other types of cause-specific deaths (S5 Table). When restricted to participants who initiated methadone between 2007 and 2015 (when custody data became available), the rate of custody admission was 0.3 per PY following methadone initiation. The subgroup analysis (S6 Table) among participants (*n* = 2,905) with at least one custody admission (AHR 0.27 [0.15–0.51]) produced findings consistent with our primary results. Similarly, the HIV cause-specific sensitivity analysis (S7 Table) produced comparable methadone treatment effects (AHR 0.19 [0.11–0.33]). Sensitivity analyses (S8 Table) involving alternate definitions for methadone periods (from 1 day to 3 and 7 days) confirm the same overall pattern of results for nonexternal (3-day AHR 0.41 [0.34–0.48]; 7-day AHR 0.52 [0.45–0.52]) and external causes (3-day AHR 0.54 [0.44–0.66); 7-day 0.59 [0.48–0.71]] of death and remain significant although, as expected, the effect decays when medicated time is inflated to 3 and 7 days.

## Discussion

In this longitudinal cohort study, dispensed methadone was associated with significantly lower risk of both all-cause and cause-specific mortality among patients diagnosed with opioid dependence and with prior convictions. To our knowledge, this is the first study to investigate the association between MMT and mortality in a large sample over an extended period (i.e., greater than 10 years) with adjustment for diverse covariates. The majority of our sample did not commit an offence in the year preceding methadone initiation (62%), and few received sentences that included time in custody (19%). Therefore, our observation period overwhelmingly corresponds to events occurring in community settings while participants were not under correctional supervision.

Our study has several implications for the treatment of opioid dependence and prevention of premature mortality in populations with complex health and social needs including exposure to corrections. The statistically significant relationship between dispensed methadone and lower risk of all-cause mortality is particularly relevant in the context of North America’s current opioid overdose crisis. The World Health Organization has recognised methadone as an essential medicine for over a decade [58] and recommended access to methadone (or other agonist treatments) for all opioid-dependent prisoners [59], acknowledging the role of untreated substance dependence as a contributor to mortality [19,60]. Despite global awareness of the importance of methadone in treating opioid dependence, a number of barriers limit the optimisation of methadone programs, including high physician patient loads [61], lack of education and training [20], and stigma [62]. These challenges are often amplified among criminal justice populations [63] and among individuals residing in remote and rural areas [64] even when offered through low-barrier services within a universal healthcare system [65]. In addition, agonist treatment options are not routinely offered alongside psychosocial or counselling interventions despite recommendations that support their importance in care [66] and their relationship to mitigating overdose risk in criminal justice populations [67]. Nevertheless, our results indicate that when methadone is administered, despite current limitations, it can reduce mortality among marginalized patients with opioid dependence. Previous research has demonstrated the substantially elevated risk of mortality among offenders during the period immediately following release from custody [13,68]. Our results expand on this work to show that mortality risk is elevated among methadone recipients with any exposure to the corrections system—where the majority are not exposed to custody—and over periods of time that greatly exceed their time under correctional supervision. Efforts to make methadone treatment more accessible, integrated, and comprehensive may yield additional life-saving benefits.

Consistent with other research [11], overdose deaths accounted for nearly one-third of mortality in our cohort. In addition to the reduced risk of all-cause mortality, our results demonstrate that adherence to methadone was associated with a lower risk of death from accidental poisoning compared with nonmedicated periods. Despite evidence that methadone adherence decreases the risk of fatal overdose [32], poor retention undermines this potential benefit. Furthermore, the potential elevated risk for overdose associated with treatment adjustments (i.e., induction and cessation) has raised concerns regarding the effectiveness of methadone as a harm-reduction measure. However, this concern is weakly supported by evidence [36] and should not be a deterrent when offering treatment with methadone because fatalities are more strongly related to other causes [35], including the illicit use of nonprescription methadone [69]. On average, participants in our cohort spent more time in nonmedicated periods than medicated periods, signalling an urgent need to substantially improve adherence.

An emerging body of evidence has highlighted the positive impact of MMT for populations with infectious disease. Patients with HIV/AIDS using methadone are associated with earlier ART initiation and higher levels of adherence [70,71]. Potential explanations are that MMT adherence is associated with increased stability and decreased risks (e.g., drug injecting), enabling increased engagement in HIV treatment. Our results are consistent with the finding that methadone may potentiate ART adherence in patients with opioid dependence, demonstrating a 5-fold lower risk of infectious disease mortality compared with nonmedicated methadone periods (in HIV subgroup analyses, this finding remained consistent).

Researchers examining deaths among offenders have focused on the risk immediately following release from custody and have consistently found significant mortality during this critical period [14]. Although the transition from prison warrants close attention to prevent mortality, a narrow focus on prison release ‘…fails to capture the ongoing elevation of risk among ex-prisoners, and directs attention away from the ongoing health needs of this chronically marginalized and unwell group’ [72] (p. 1555). Current harm reduction and addictions literature advocates for the treatment and management of substance misuse as a chronic disease [40] rather than an acute episodic illness requiring detoxification [73]. This approach is supported by observations that a majority of opioid-dependent individuals receiving methadone have repeated treatment episodes, with continuous and/or longer treatment duration typically occurring after several failed attempts [74]. The paucity of literature evaluating mortality risk over extended time periods limits our understanding of risk related to the chronic and relapsing nature of opioid dependence among individuals with criminal justice involvement. Our research design aimed to address this gap by investigating treatment as it fluctuates over a relatively long follow-up period.

Our study offers the advantages of a complete population of convicted offenders accessing methadone, with specific objective measurement of exposure and outcome while controlling for several key covariates. Our sample included individuals who were exposed to provincial corrections, typically for short periods of time, serving sentences in noncustodial settings. Therefore, our observation period overwhelmingly represents community-based methadone prescribing. Despite its strengths, this study has limitations. The use of a Canadian provincial offender population may limit the generalizability of results to other settings, jurisdictions, and patient groups. Our outcome is restricted to recorded deaths and does not account for undetected mortality. Also, our findings may have been subject to compliance bias because adherence to MMT may indicate unmeasured behaviours that, in turn, have an influence on mortality. The magnitude of the protective effects of methadone from nonexternal causes of death should be interpreted with caution because our results and analyses do not take into account methadone prescribed in hospitals or other care facilitates (e.g., hospice); however, this limitation does not affect the magnitude of effect for external causes of mortality. Receipt of methadone treatment may be accompanied by psychosocial supports (e.g., counselling supports, Alcoholics Anonymous [AA], Narcotics Anonymous [NA], etc.) with varying degrees of participation by individuals. Involvement with ancillary supports was not accounted for in our analyses and may have altered treatment adherence. Methadone prescribing in BC almost universally involves witnessed methadone ingestion, and therefore our use of pharmacy dispensing records provides a strong basis for inferring methadone adherence. Disruptions to treatment, such as access or relocation, were not assessed and may have influenced our results. The influence of other opioid prescriptions (buprenorphine and buprenorphine-naloxone) prior to or during the observation period was minimal (approximately 1% of the total opiate agonist prescriptions between 2008 and 2015); however, their exclusion is a limitation to our study. Lastly, we did not account for methadone dose, which has been shown to be related to mortality outcomes [75]; therefore, research examining the relationship between dose and mortality is needed, as is research investigating additional opiate treatments (e.g., suboxone).

## Conclusion

In a large cohort of Canadian convicted offenders, rates of mortality were significantly lower during periods when individuals were dispensed methadone compared with periods in which they were not dispensed methadone. Our findings strongly indicate that efforts to increase methadone adherence may reduce mortality in high-risk populations such as opioid-dependent offenders. Our findings warrant examination in other study centres in response to the crisis of opiate-involved deaths.

## Supporting information

S1 Text S1 Statistical Analysis Plan—for “Methadone Maintenance Treatment and Mortality in People with Criminal Convictions: A Population-based Retrospective Cohort Study from Canada”. (DOCX)

S2 Text RECORD checklist. (DOCX)

S1 Table Sociodemographic, methadone-related, and other characteristics among 1,275 deceased offenders from BC, 1998–2015. BC, British Columbia.
(DOCX)

S2 Table AHR estimates of methadone and other predictors on all-cause mortality among 14,530 convicted offenders from BC, 1998–2015. AHR, adjusted hazard ratio.
(DOCX)

S3 Table AHR estimates of methadone and other predictors on nonexternal cause-specific mortality among convicted 14,530 offenders from BC, 1998–2015. AHR, adjusted hazard ratio.
(DOCX)

S4 Table AHR estimates of methadone and other predictors on external cause-specific mortality among 14,530 convicted offenders from BC, 1998–2015. AHR, adjusted hazard ratio.
(DOCX)

S5 Table Competing-risks regression analysis to estimate sub-HR of dispensed methadone (versus nondispensed methadone) on cause-specific mortality among 14,530 convicted offenders from BC, 1998–2015. BC, British Columbia; HR, hazard ratio.
(DOCX)

S6 Table AHR estimates of medicated methadone and other predictors on all-cause deaths among 7,722 convicted offenders from BC, 2007–2015. AHR, adjusted hazard ratio.
(DOCX)

S7 Table AHR estimates of medicated methadone and other predictors on HIV-related deaths among 14,530 convicted offenders from BC, 1998–2015. AHR, adjusted hazard ratio; BC, British Columbia.
(DOCX)

S8 Table Sensitivity analysis to estimate the effect of methadone on mortality with inflated time on last methadone fill among 14,530 convicted offenders from BC, 1998–2015. (DOCX)

## Abbreviations

AA: Alcoholics Anonymous
AHR: adjusted HR
ART: antiretroviral treatment
BC: British Columbia
DSM: Diagnostic and Statistical Manual of Mental Disorders
HR: hazard ratio
IMRI: Inter-Ministry Research Initiative
IQR: interquartile range
ICD-10: International Statistical Classification of Diseases and Related Health Problems 10th Revision
MMT: methadone maintenance treatment
NA: Narcotics Anonymous
OST: opioid substitution treatment
UHR: unadjusted HR
PY: person-year
