# Supplementary material for: Methadone maintenance treatment and mortality in people with criminal convictions: A population-based retrospective cohort study from Canada
Source: PLoS Med. 2018 Jul 31;15(7):e1002625. doi: 10.1371/journal.pmed.1002625 (PMC6067717; doi:10.1371/journal.pmed.1002625)
Supplement: S1 Text — (DOCX) [file pmed.1002625.s001.docx]

**S1 Statistical Analysis Plan - for “*Methadone Maintenance Treatment and Mortality in People with Criminal Convictions: A Population-based Retrospective Cohort Study from Canada”***

**Analysis Summary**:

- No prospective protocol was published or registered for this cohort study. We did however, follow a clear analysis plan as described in the methods section and did not deviate from this plan.
- The inclusion/exclusion criteria for this study were established at the outset, based on related previously published research and were not changed. A description of this process is included in Figure 1 of the manuscript.
- Definitions for variables were established at the outset and were not changed. Individual time intervals were calculated using pharmacy filing transaction dates. If a participant filled their methadone prescription consistently (no gap in pharmacy transactions dates) for a period of time, this was treated as a single interval/episode and considered as a medicated period (methadone was dispensed). If the participant didn’t fill a prescription for a period of time (gap in pharmacy transaction dates), the interval was considered a non-medicated period (methadone was not dispensed). The main outcome was death during follow-up. Causes of death were coded according to the International Statistical Classification of Diseases and Related Health Problems 10^th^ Revision (ICD-10
- Data was pre-specified at the outset and was not altered. Data were obtained by linking population-level administrative records in British Columbia (BC) Canada under the Inter-Ministry Research Initiative (IMRI). The IMRI serves as a resource for the development of policies and services that span health, justice, and social welfare sectors. Data on the main exposure, methadone, were extracted from the PharmaNet database, a province-wide network linking all prescriptions issued by BC pharmacies.
- The main statistical analyses were determined at the outset and were not changed. We planned to compare the rates of death during periods when participants were dispensed methadone treatment with periods in which they were not dispensed methadone treatment. We hypothesized an increase in all cause and overdose deaths during periods when individuals were off treatment. We also assessed the adjusted hazard ratio estimates of methadone on all-cause, non-external and external causes of death and included a competing risk regression to estimate the sub-hazard ration of dispensed methadone on cause-specific mortality.
- Details on our methods for how time intervals for methadone were constructed and accounted for in the Cox regression model are noted below on page 2 of this document.
- Our outcome - death – was determined at the outset and was not altered.
- We conducted a number of additional analyses following reviewers feedback in April 2018 (unless otherwise indicated) which included:
  - A sensitivity analyses inflating the last MMT medicated time by 3 and 7 days
  - A sensitivity analyses excluding the time-dependent covariate "#offences from MMT-start"
  - Models that included gender by age-group interactions as well as methadone treatment by age-group interactions for all-cause mortality and accidental poisoning.;
  - A subgroup analysis among participants (who initiated methadone between Jan 2007 & Mar 2015) with additional time-varying covariates (number of custody admissions since methadone initiation)
  - A separate cause specific analysis for HIV related deaths; we conducted a sensitivity analysis restricting follow-up to 1 & 2 years for all-cause mortality.
  - Descriptive statistics describing time leading up to death for individuals who died during the study period (May 2018)

**Methods for how time intervals for methadone were constructed and accounted for in the Cox regression model**

In the current paper, we have chosen time-to-event/ survival analysis since our outcome of interest (death) was not only occurrence of event, but also when the event occurred. Cox regression is the most widely used time-to-event analysis in health research. Moreover, censoring was another important consideration, favouring time-to-event analysis. In the presence of censoring, exact time-to-event is undeterminable. Traditional regression analysis is not well equipped to incorporate both occurrence of event and time-to-event as an outcome. In addition, our analysis was complicated due to the time varying (values change over time) nature of the primary independent variable (methadone status: medicated vs. non-medicated period). Data set-up for survival analysis with time-varying covariate is an important consideration to conduct the regression. The time varying covariates can be incorporated in the time-to-event analysis by sub-dividing the follow-up time into intervals/segments. This data structure procedure is referred to as counting process (CP). This process essentially subdivides an individual’s entire time at risk (follow-up time) into smaller time segments to absorb the change of values of time-dependent covariates, resulting in multiple observations per participant. This document is intended to illustrate how time intervals for methadone are constructed and accounted for in the Cox regression model. We have chosen four hypothetical participants to describe this procedure.

Individual time intervals were calculated using pharmacy filing transaction dates. If a participant filled their methadone prescription consistently (no gap in pharmacy transactions dates) for a period of time, this was treated as a single interval/episode and considered as a medicated period (methadone was dispensed). If the participant didn’t fill a prescription for a period of time (gap in pharmacy transaction dates), the interval was considered a non-medicated period (methadone was not dispensed). Therefore, participants were continuously alternating between medicated and non-medicated episodes during the observation period.

For example, Participant A initiated methadone on 1-Jan-14 and filled their methadone prescription continuously (no gap) until 31-Jan-14 (this can be a single pharmacy transaction with 30 days of drug supply or 30 pharmacy transactions with single day of drug supply for each transaction). Following 31-Jan-14, participant A didn’t have any pharmacy transactions until 28-Feb-14. For this participant, January was considered a medicated time interval with duration of 30 days and February was considered a non-medicated time interval with duration of 28 days. After February, this participant received methadone for another 89 days (28-Feb-14 to 28-May-14) followed by two days off-treatment (28-May-14 to 30-May-14). This participant died on 18-Mar-15 and was censored on that day. Total follow-up time for participant A was 441 days (1-Jan-14 to 18-Mar-15), which included 12 time-intervals (six medicated and six non-medicated intervals). Due to time varying nature of methadone medication status, this participant (A) yielded 12 observations. This participant (A) did experience failure (death) during non-medicated period, the mortality rate was: overall (1/441 days, 0.8 per PYs), medicated period (0/260 days, 0.0 per PYs) and non-medicated period (1/181 days, 2.0 per PYs).

Participant B initiated methadone on 10-Sep-14 and was censored on 10-Dec-14 due to death. This participant, who was another example of failure, contributed only 91 analysis-days, mostly medicated time (84 days vs. 7 days) with 11 time intervals (six medicated and five non-medicated intervals). The overall mortality rate was 4.0 per PYs (1/91 days). However, this participant (B) died during the medicated period (dissimilar to A), the mortality rate for medicated period was 1/84 days, 4.3 per PYs and non-medicated period was 0/7 days, 0.0 per PYs.

**Table 1A: Data structure of primary exposure variable (methadone) for the cox regression for four hypothetical participants**

| id | rx | sdt | edt | dur | time0 | time1 | event | gender |
| --- | --- | --- | --- | --- | --- | --- | --- | --- |
| A | 1 | 1-Jan-14 | 31-Jan-14 | 30 | 0 | 30 | 0 | M |
| A | 0 | 31-Jan-14 | 28-Feb-14 | 28 | 30 | 58 | 0 | M |
| A | 1 | 28-Feb-14 | 28-May-14 | 89 | 58 | 147 | 0 | M |
| A | 0 | 28-May-14 | 30-May-14 | 2 | 147 | 149 | 0 | M |
| A | 1 | 30-May-14 | 30-Jun-14 | 31 | 149 | 180 | 0 | M |
| A | 0 | 30-Jun-14 | 1-Sep-14 | 63 | 180 | 243 | 0 | M |
| A | 1 | 1-Sep-14 | 30-Sep-14 | 29 | 243 | 272 | 0 | M |
| A | 0 | 30-Sep-14 | 30-Nov-14 | 61 | 272 | 333 | 0 | M |
| A | 1 | 30-Nov-14 | 30-Jan-15 | 61 | 333 | 394 | 0 | M |
| A | 0 | 30-Jan-15 | 8-Feb-15 | 9 | 394 | 403 | 0 | M |
| A | 1 | 8-Feb-15 | 28-Feb-15 | 20 | 403 | 423 | 0 | M |
| **A** | **0** | **28-Feb-15** | **18-Mar-15** | **18** | **423** | **441** | **1** | **M** |
|  |  |  |  |  |  |  |  |  |
| B | 1 | 10-Sep-14 | 23-Sep-14 | 13 | 0 | 13 | 0 | M |
| B | 0 | 23-Sep-14 | 24-Sep-14 | 1 | 13 | 14 | 0 | M |
| B | 1 | 24-Sep-14 | 13-Oct-14 | 19 | 14 | 33 | 0 | M |
| B | 0 | 13-Oct-14 | 14-Oct-14 | 1 | 33 | 34 | 0 | M |
| B | 1 | 14-Oct-14 | 20-Oct-14 | 6 | 34 | 40 | 0 | M |
| B | 0 | 20-Oct-14 | 21-Oct-14 | 1 | 40 | 41 | 0 | M |
| B | 1 | 21-Oct-14 | 22-Oct-14 | 1 | 41 | 42 | 0 | M |
| B | 0 | 22-Oct-14 | 24-Oct-14 | 2 | 42 | 44 | 0 | M |
| B | 1 | 24-Oct-14 | 29-Oct-14 | 5 | 44 | 49 | 0 | M |
| B | 0 | 29-Oct-14 | 31-Oct-14 | 2 | 49 | 51 | 0 | M |
| **B** | **1** | **31-Oct-14** | **10-Dec-14** | **40** | **51** | **91** | **1** | **M** |
|  |  |  |  |  |  |  |  |  |
| C | 1 | 15-Dec-12 | 31-Dec-12 | 16 | 0 | 16 | 0 | F |
| C | 0 | 31-Dec-12 | 24-Jan-13 | 24 | 16 | 40 | 0 | F |
| C | 1 | 24-Jan-13 | 16-May-13 | 112 | 40 | 152 | 0 | F |
| C | 0 | 16-May-13 | 19-Sep-13 | 126 | 152 | 278 | 0 | F |
| C | 1 | 19-Sep-13 | 19-Dec-13 | 91 | 278 | 369 | 0 | F |
| C | 0 | 19-Dec-13 | 2-Feb-14 | 45 | 369 | 414 | 0 | F |
| C | 1 | 2-Feb-14 | 5-Jun-14 | 123 | 414 | 537 | 0 | F |
| C | 0 | 5-Jun-14 | 2-Jan-15 | 211 | 537 | 748 | 0 | F |
| C | 1 | 2-Jan-15 | 2-Feb-15 | 31 | 748 | 779 | 0 | F |
| C | 0 | 2-Feb-15 | 3-Feb-15 | 1 | 779 | 780 | 0 | F |
| C | 1 | 3-Feb-15 | 31-Mar-15 | 56 | 780 | 836 | 0 | F |
|  |  |  |  |  |  |  |  |  |
| D | 1 | 31-Mar-14 | 31-Mar-15 | 365 | 0 | 365 | 0 | M |

Id: identifier variable; rx: methadone (0-non-medicated status, 1-medicated status); sdt: start date of each time-interval; edt: end date of each time-interval; dur: duration of each time-interval; time0: start time of each time-interval, in days; time1: end time of each time-interval, in days; event: outcome of interest (0-not occurred, 1-occurred); gender: Male (M) & Female (F)

Participant C initiated methadone on 15-Dec-12 and was censored on 31-Mar-15 when the study ended. Participant C had a long follow-up time of 836 days, but had only 11 time intervals (six medicated and five non-medicated intervals). Since this participant didn’t experience any failure (death), the death rate was zero for all instances: overall (0/836 days, 0.0 per PYs), medicated period (0/429 days, 0.0 per PYs) and non-medicated period (0/407 days, 0.0 per PYs).

Participant D initiated methadone on 31-Mar-14 and did continue receiving methadone until the end of study (March 31, 2015). This participant didn’t have any gap in dispensing methadone, which resulted one medicated time-interval (no non-medicated time interval) as well as single observation. This participant was another example of non-failure (alive). Therefore, the mortality rate was zero for all instances: overall (0/365 days, 0.0 per PYs), medicated period (0/365 days, 0.0 per PYs) and non-medicated period (0/0 days, 0.0 per PYs).

**Table 1B: Summary statistic for four hypothetical participants (A, B, C & D)**

|  | **Participant A** | **Participant B** | **Participant C** | **Participant D^[[1]](#footnote-1)^** |
| --- | --- | --- | --- | --- |
| Censoring | Censored on March 18, 2015 (due to death) | Censored on 10-Dec-14 (due to death) | Censored on March 31, 2015 (study end) | Censored on March 31, 2015 (study end) |
| # of time intervals | 12 | 11 | 11 | 1 |
| # of medicated intervals | 6 | 6 | 6 | 1 |
| # of non-medicated intervals | 6 | 5 | 5 | 0 |
| Total follow-up/analysis time, days | 441 | 91 | 836 | 365 |
| Total medicated time, days | 260 | 84 | 429 | 365 |
| Total non-medicated time, days | 181 | 7 | 407 | 0 |
| Longest medicated interval, days | 89 | 40 | 123 | 365 |
| Longest non-medicated interval, days | 63 | 2 | 211 | 0 |
| Number of events | 1 | 1 | 0 | 0 |
| Death rate, per person-year  Overall  On Methadone (medicated period)  Off Methadone (non-medicated period) | 0.8  0.0  2.0 | 4.0  4.3  0.0 | 0.0  0.0  0.0 | 0.0  0.0  0.0 |

Altogether, these four participants (A, B, C & D) had 35 time-intervals or observations (19 medicated periods and 16 non-medicated periods) and contributed 1,733 follow-up days (4.8 PYs) with two failures. The mortality rate: overall (2/1733 days, 0.4 per PYs), medicated period (1/1138 days, 0.3 per PYs) and non-medicated period (1/595 days, 0.6 per PYs).

**Table 2A: ICD-9 Diagnostic codes (290-319) for Mental disorders^[[2]](#footnote-2)^**

|  | **CODE** | **Description of disorder** |
| --- | --- | --- |
| **ICD-9** | 290 | SENILE AND PRESENILE ORGANIC PSYCHOTIC CONDITIONS |
|  | 291* | *ALCOHOLIC PSYCHOSES* |
|  | 292* | *DRUG PSYCHOSES* |
|  | 293 | TRANSIENT ORGANIC PSYCHOTIC CONDITIONS |
|  | 294 | OTHER ORGANIC PSYCHOTIC CONDITIONS (CHRONIC) |
|  | 295 | SCHIZOPHRENIC PSYCHOSES |
|  | 296 | AFFECTIVE PSYCHOSES |
|  | 297 | PARANOID STATES |
|  | 298 | OTHER NONORGANIC PSYCHOSES |
|  | 299 | PSYCHOSES WITH ORIGIN SPECIFIC TO CHILDHOOD |
|  | 300 | NEUROTIC DISORDERS |
|  | 301 | PERSONALITY DISORDERS |
|  | 302 | SEXUAL DEVIATIONS AND DISORDERS |
|  | 303* | *ALCOHOL DEPENDENCE SYNDROME* |
|  | 304* | *DRUG DEPENDENCE* |
|  | 305* | *NONDEPENDENT ABUSE OF DRUGS* |
|  | 306 | PHYSIOLOGICAL MALFUNCTION ARISING FROM MENTAL FACTORS |
|  | 307 | SPECIAL SYMPTOMS OR SYNDROMES NOT ELSEWHERE CLASSIFIED |
|  | 308 | ACUTE REACTION TO STRESS |
|  | 309 | ADJUSTMENT REACTION |
|  | 310 | SPECIFIC NONPSYCHOTIC MENTAL DISORDERS FOLLOWING ORGANIC BRAIN DAMAGE |
|  | 311 | DEPRESSIVE DISORDER, NOT ELSEWHERE CLASSIFIED |
|  | 312 | DISTURBANCE OF CONDUCT NOT ELSEWHERE CLASSIFIED |
|  | 313 | DISTURBANCE OF EMOTIONS SPECIFIC TO CHILDHOOD AND ADOLESCENCE |
|  | 314 | HYPERKINETIC SYNDROME OF CHILDHOOD |
|  | 315 | SPECIFIC DELAYS IN DEVELOPMENT |
|  | 316 | PSYCHIC FACTORS ASSOCIATED WITH DISEASES CLASSIFIED ELSEWHERE |
|  | 317 | MILD MENTAL RETARDATION |
|  | 318 | OTHER SPECIFIED MENTAL RETARDATION |
|  | 319 | UNSPECIFIED MENTAL RETARDATION |
|  | 50B | Anxiety/Depression |

ICD: INTERNATIONAL CLASSIFICATION OF DISEASES

* Indicates the diagnostic codes that were used to ascertain Substance Use Disorders (SUD).

**3A: Description of methadone and controlling variables included in the multivariable cox regression**

| ***Name of Variables*** | **Time of measurement** | **Analytic type & levels** | **Time varying** | **Reference level** |
| --- | --- | --- | --- | --- |
| ***Methadone*** | During the follow up period (Initiation to death or study end, march 31, 2015) | Binary (medicated & non-medicated) | Yes | Non-medicated period |
| ***Age*** | At the time of methadone initiation | Categorical with five levels  <25 years, 25 to 34 years, 35 to 44 years, 45 to 54 years & 55 years or older | No | <25 years |
| ***Gender*** | Self-reported, time of justice contact | Binary (men & women) | No | Women |
| ***Ethnicity*** | Self-reported, time of justice contact | Categorical with three levels  White, Indigenous & Other  Unknown included as separate level | No | Other |
| ***Education level*** | Self-reported, time of justice contact | Categorical with four levels  <Grade 10, Grade 10/11, Grade 12 & Vocational /University  Unknown included as separate level | No | Vocational /University |
| ***Year of methadone initiation*** | Calendar year of methadone initiation | Categorical with four levels  1998 to 2000, 2001 to 2005, 2006 to 2010, 2011 to 2015^[[3]](#footnote-3)^ | No | 1998 to 2000 |
| ***Any offence*** | In the one-year period prior to methadone initiation | Categorical with three levels  None, 1-2 offences & > 2 offences | No | No offence |
| ***# of offences after methadone initiation*** | During the follow up period (Initiation to death or study end, march 31, 2015) | Continuous | Yes | Per crime |
| ***# of custody admissions after methadone initiation^[[4]](#footnote-4)^*** | During the follow up period between Jan 2007 and Mar 2015 (Initiation to death or study end, march 31, 2015) | Continuous | Yes | Per admission |
| ***Severe mental illness*** | Ever | Categorical with three levels No Schizophrenia/Bipolar,  Schizophrenia^[[5]](#footnote-5)^ & Bipolar | No | No Schizophrenia/Bipolar |
| ***MSP services (NSMD^[[6]](#footnote-6)^ related)*** | In the five-year period prior to methadone initiation | Categorical with three levels  Low^[[7]](#footnote-7)^ (≤ 2), Medium (3 to 10) & High (≥11) | No | Low (≤ 2) |
| ***MSP services (SUD^[[8]](#footnote-8)^ related)*** | In the five-year period prior to methadone initiation | Categorical with three levels  Low^[[9]](#footnote-9)^ (≤ 4), Medium (5 to 13) & High (≥14) | No | Low (≤ 4) |
| ***MSP services (non-psychiatric)*** | In the five-year period prior to methadone initiation | Categorical with three levels  Low^[[10]](#footnote-10)^ (≤ 69), Medium (70 to 139) & High (≥140) | No | Low (≤ 69) |

MSP: Medical Service Plan; NSMD: Non-Substance Mental Disorder; SUD: Substance Use Disorder

1. -Participant D, who was an extreme example of methadone medication status, was considered here for illustrative purposes. [↑](#footnote-ref-1)
2. -This table includes description of disorders as listed in the document accessed on May 18, 2016. URL: <http://www2.gov.bc.ca/assets/gov/health/practitioner-pro/medical-services-plan/diag-codes_mental.pdf> [↑](#footnote-ref-2)
3. -2015 included only three months (January to March) of data [↑](#footnote-ref-3)
4. -Restricted to participants who initiated between Jan 2007 and Mar 2015. [↑](#footnote-ref-4)
5. -Three digits ICD-9 code 295 and 296 were used to ascertain Schizophrenia and Bipolar respectively. [↑](#footnote-ref-5)
6. - Non-substance mental disorder (NSMD) consisted of ICD-9 diagnostic codes (290-319) for Mental disorders (except 291, 292, 303, 304, and 305) and 50B (Anxiety/Depression). [↑](#footnote-ref-6)
7. - 50^th^ & 75^th^ percentile was used to categorize into low, medium and high groups. [↑](#footnote-ref-7)
8. - Substance use disorders (SUD) were identified using the three-digit codes of 291, 292, 303, 304, and 305. [↑](#footnote-ref-8)
9. -50^th^ & 75^th^ percentile was used to categorize into low, medium and high groups [↑](#footnote-ref-9)
10. -50^th^ & 75^th^ percentile was used to categorize into low, medium and high groups [↑](#footnote-ref-10)
