# Supplementary material for: Methadone maintenance treatment and mortality in people with criminal convictions: A population-based retrospective cohort study from Canada
Source: PLoS Med. 2018 Jul 31;15(7):e1002625. doi: 10.1371/journal.pmed.1002625 (PMC6067717; doi:10.1371/journal.pmed.1002625)
Supplement: S1 Table — BC, British Columbia. (DOCX) [file pmed.1002625.s003.docx]

**S1 Table: Socio-demographics, methadone and other related characteristics among 1,275 deceased offenders from British Columbia, 1998 to 2015**

|  | **All-cause mortality (n=1,275)**  **mean (SD)/ n (%)** | **Non-external cause mortality (n=771)**  **mean (SD)/ n (%)** | **External cause mortality (n=504)**  **mean (SD)/ n (%)** |
| --- | --- | --- | --- |
| ***Age groups (years), n (%)***  18 < 25  25 < 35  35 < 45  45 < 55  ≥ 55 | 117 (9.2)  333 (26.1)  455 (35.7)  286 (22.4)  84 (6.6) | 51 (6.6)  160 (20.8)  274 (35.5)  218 (28.3)  68 (8.8) | 66 (13.1)  173 (34.3)  181 (35.9)  68 (13.5)  16 (3.2) |
| **Men, n%** | 948 (74.3) | 562 (72.9) | 386 (76.6) |
| ***Ethnicity, n (%)***  White  Indigenous  Other  Unknown | 984 (77.2)  188 (14.8)  72 (5.6)  31 (2.4) | 598 (77.6)  120 (15.6)  32 (4.1)  21 (2.7) | 386 (76.6)  68 (13.5)  40 (7.9)  10 (2.0) |
| ***Education level, n (%)***  <Grade 10  Grade 10/11  Grade 12  Vocational /University  Unknown | 175 (13.7)  456 (35.8)  444 (34.8)  142 (11.1)  58 (4.6) | 104 (13.5)  283 (36.7)  264 (34.2)  78 (10.1)  42 (5.5) | 71 (14.1)  173 (34.3)  180 (35.7)  64 (12.7)  16 (3.2) |
| ***Medicated treatment period, in days***  Mean (SD)  Median (IQR) | 1076.0 (1191.3)  637 (155, 1630) | 1224.7 (1262.9)  846 (183, 1898) | 848.6 (1033.3)  447 (119, 1230) |
| ***Number of medicated treatment period/episode***  Mean (SD)  Median (IQR) | 37.2 (48.0)  20 (6.48) | 40.3 (50.4)  23 (7, 53) | 32.6 (43.7)  17 (5. 40) |
| ***Non-medicated treatment period, in days***  Mean (SD)  Median (IQR) | 1083.1 (1155.6)  668 (175, 1620) | 1099.1 (1190.8)  712 (143, 1672) | 1058.5 (110.3)  630 (210, 1553) |
| ***Number of non-medicated treatment period/episode***  Mean (SD)  Median (IQR) | 37.0 (48.0)  20 (6.48) | 40.1 (50.5)  22 (7, 53) | 32.4 (43.7)  17 (5. 40) |
| ***Treatment status during last period prior to death, n (%)***  Non-Medicated period  Medicated period | 996 (78.1)  279 (21.9) | 623 (80.8)  148 (19.2) | 373 (74.0)  131 (26.0) |
| ***Duration of last non-medicated period in days***  Mean (SD)  Median (IQR) | 731.2 (1046.6)  250 (29, 1047) | 669.9 (1043.8)  122 (19, 954) | 833.6 (1044.7)  404 (106, 1188) |
| ***Duration of last medicated period in days***  Mean (SD)  Median (IQR**)** | 97.3 (195.7)  25 (6, 97) | 109.5 (232.0)  28 (7, 105) | 83.6 (143.9)  16 (4, 88) |
| ***Year of death, n (%)***  1998 to 2000  2001 to 2005  2006 to 2010  2011 to 2015^[[1]](#footnote-1)^ | 18 (1.4)  258 (20.2)  445 (34.9)  554 (43.5) | 13 (1.7)  126 (16.3)  258 (33.5)  374 (48.5) | 5 (1.0)  132 (26.2)  187 (37.1)  180 (35.7) |
| ***Severe mental illness, n (%)***  No Schizophrenia or Bipolar  Schizophrenia  Bipolar | 852 (66.8)  189 (14.8)  234 (18.4) | 547 (71.0)  89 (11.5)  135 (17.5) | 305 (60.5)  100 (19.9)  99 (19.6) |
| ***Any offence in the year prior to enrolment, n (%)***  None  1-2 offences  > 2 offences | 794 (62.3)  295 (23.1)  186 (14.6) | 484 (62.8)  178 (23.1)  109 (14.1) | 310 (61.5)  117 (23.2)  77 (15.3) |
| ***# of hospitalizations^[[2]](#footnote-2)^***  Mean (SD)  Median (IQR) | 9.6 (10.3)  7 (3, 13) | 10.9 (11.2)  8 (4, 15) | 7.5 (8.5)  5 (2, 9) |
| ***History of any hospitalizations^[[3]](#footnote-3)^, n (%)*** | 1,227 (96.2) | 763 (99.0) | 464 (92.1) |
| ***Death since last discharge from hospitals^[[4]](#footnote-4)^ (in days), n (%)***  Same day^[[5]](#footnote-5)^  1-7 days  8-30 days  31-180 days  > 180 days | 499 (40.8)  53 (4.3)  73 (6.0)  158 (12.9)  441 (36.0) | 441 (57.9)  34 (4.5)  53 (7.0)  84 (11.0)  150 (19.7) | 58 (12.6)  19 (4.1)  20 (4.3)  74 (16.0)  291 (63.0) |
| **Length of stay during last (prior to death) hospitalization**  Mean (SD)  Median (IQR) | 11.6 (24.9)  4 (1, 13) | 15.1 (29.6)  6 (2, 18) | 5.8 (12,2)  2 (1, 6) |
| ***# of incarcerations^[[6]](#footnote-6)^***  Mean (SD)  Median (IQR) | 1.7 (3.1)  0 (0, 2) | 1.8 (3.5)  0 (0, 2) | 1.6 (2.6)  0 (0, 2) |
| ***History of any incarcerations^[[7]](#footnote-7)^, n (%)*** | 142 (44.2) | 83 (44.4) | 59 (44.0) |
| ***Death since last release from prison^[[8]](#footnote-8)^ (in days), n (%)***  Same day^[[9]](#footnote-9)^  1-7 days  8-30 days  31-180 days  > 180 days | 4 (2.8)  8 (5.6)  7 (4.9)  24 (16.9)  99 (69.7) | 3 (3.6)  1 (1.2)  3 (3.6)  11 (13.3)  65 (78.3) | 1 (1.7)  7 (11.9)  4 (6.8)  13 (22.0)  34 (57.6) |

1. -2015 included only three months (January to March) of data [↑](#footnote-ref-1)
2. -Indicates time period between January 1990 to March 2015. [↑](#footnote-ref-2)
3. -Indicates time period between January 1990 to March 2015. [↑](#footnote-ref-3)
4. -Restricted to participants who died and had a history of hospitalizations (all-cause: n=1,224; non-external cause: n=762 & external cause: n=462). Out of 1,227 cases, three participants were excluded from denominator due to date error. [↑](#footnote-ref-4)
5. -Participants died in hospitals (most likely). [↑](#footnote-ref-5)
6. -Restricted to participants who died and initiated methadone between January 2007 to March 2015 (all-cause: n=321; non-external cause: n=187 & external cause: n=134). Indicates time period between January 2007 to March 2015. [↑](#footnote-ref-6)
7. -Restricted to participants who died and initiated methadone between January 2007 to March 2015 (n=321). Indicates time period between January 2007 to March 2015. [↑](#footnote-ref-7)
8. -Restricted to participants who initiated methadone between January 2007 to March 2015 and also had a history of incarceration (all-cause: n=142; non-external cause: n=83 & external cause: n=59). [↑](#footnote-ref-8)
9. -Participants died in prison (most likely). [↑](#footnote-ref-9)
