# Supplementary material for: Methadone maintenance treatment and mortality in people with criminal convictions: A population-based retrospective cohort study from Canada
Source: PLoS Med. 2018 Jul 31;15(7):e1002625. doi: 10.1371/journal.pmed.1002625 (PMC6067717; doi:10.1371/journal.pmed.1002625)
Supplement: S2 Table — AHR, adjusted hazard ratio. (DOCX) [file pmed.1002625.s004.docx]

**S2 Table: AHR estimates of methadone and other predictors on all-cause mortality among 14,530 convicted offenders from BC, 1998–2015. AHR, adjusted hazard ratio.**

| **Variables** | **All-cause mortality (n=1,275)**  **AHR (95% CI)** |
| --- | --- |
| ***Methadone (medicated period)*** | **0.32 (0.28, 0.37)** |
| ***Age groups (years)***  18 < 25  25 < 35  35 < 45  45 < 55  ≥ 55 | Reference  **1.40 (1.13, 1.73)^[[1]](#footnote-1)^**  **2.46 (2.00, 3.04)**  **4.51 (3.58, 5.67)**  **10.06 (7.41, 13.67)** |
| ***Men (vs. Women)*** | **1.17 (1.02, 1.35)** |
| ***Ethnicity***  White  Indigenous  Other  Unknown | **1.40 (1.10, 1.80)**  **1.42 (1.07, 1.88)**  Reference  1.50 (0.92, 2.43) |
| ***Education level***  <Grade 10  Grade 10/11  Grade 12  Vocational /University  Unknown | 1.13 (0.90, 1.42)  **1.37 (1.13, 1.66)**  1.19 (0.98, 1.44)  Reference  0.94 (0.66, 1.34) |
| ***Year of methadone initiation***  1998 to 2000  2001 to 2005  2006 to 2010  2011 to 2015^[[2]](#footnote-2)^ | Reference  0.96 (0.84, 1.11)  **0.83 (0.70, 0.98)**  0.86 (0.68, 1.09) |
| ***Any offence in the year prior to enrolment***  None  1-2 offences  > 2 offences | Reference  1.05 (0.92, 1.21)  1.04 (0.88, 1.23) |
| ***# of offences after enrolment, per offence*** | 1.00 (1.00, 1.01) |
| ***Severe mental illness***  No Schizophrenia or Bipolar  Schizophrenia  Bipolar | **Reference**  0.90 (0.76, 1.07)  0.93 (0.80, 1.08) |
| ***MSP services (NSMD related) in the five-year period prior to enrolment***  Low^[[3]](#footnote-3)^ (≤ 2)  Medium (3 to 10)  High (≥11) | Reference  1.12 (0.96, 1.30)  **1.24 (1.04, 1.47)** |
| ***MSP services (SUD related) in the five-year period prior to enrolment***  Low^[[4]](#footnote-4)^ (≤ 4)  Medium (5 to 13)  High (≥14) | Reference  **1.24 (1.08, 1.42)**  1.03 (0.89, 1.19) |
| ***MSP services (non-psychiatric) in the five-year period prior to enrolment***  Low^[[5]](#footnote-5)^ (≤ 69)  Medium (70 to 139)  High (≥140) | Reference  **1.25 (1.07, 1.46)**  **1.74 (1.47, 2.06)** |

AHR: Adjusted Hazard Ratio; CI: Confidence Interval; MSP: Medical Services Plan; NSMD: Non-Substance Mental Disorder; SUD: Substance Use Disorder

1. -Bold indicates significance of HR (p <0.05) [↑](#footnote-ref-1)
2. -2015 included only three months (January to March) of data [↑](#footnote-ref-2)
3. -50^th^ & 75^th^ percentile was used to categorize into low, medium and high groups. [↑](#footnote-ref-3)
4. -50^th^ & 75^th^ percentile was used to categorize into low, medium and high groups [↑](#footnote-ref-4)
5. -50^th^ & 75^th^ percentile was used to categorize into low, medium and high groups [↑](#footnote-ref-5)
