# Supplementary material for: Methadone maintenance treatment and mortality in people with criminal convictions: A population-based retrospective cohort study from Canada
Source: PLoS Med. 2018 Jul 31;15(7):e1002625. doi: 10.1371/journal.pmed.1002625 (PMC6067717; doi:10.1371/journal.pmed.1002625)
Supplement: S3 Table — AHR, adjusted hazard ratio. (DOCX) [file pmed.1002625.s005.docx]

**S3 Table: AHR estimates of methadone and other predictors on nonexternal cause-specific mortality among convicted 14,530 offenders from BC, 1998–2015. AHR, adjusted hazard ratio.**

| **Variables** | **1: All non-external cause mortality (n=771)**  **AHR (95% CI)** | **1A: Infectious diseases (n=190)**  **AHR (95% CI)** | **1B: Other non-external causes (n=581)**  **AHR (95% CI)** |
| --- | --- | --- | --- |
| ***Methadone (medicated period)*** | **0.27 (0.23, 0.33)** | **0.20 (0.13, 0.30)** | **0.30 (0.25, 0.37)** |
| ***Age groups (years)***  18 < 25  25 < 35  35 < 45  45 < 55  ≥ 55 | Reference  **1.63 (1.18, 2.25)**  **3.7 (2.72, 5.03)**  **8.78 (6.37, 12.1)**  **20.05 (13.53, 29.71)** | Reference  **2.08 (1.14, 3.81)**  **4.84 (2.74, 8.56)**  **9.41 (5.15, 17.16)**  **6.38 (2.02, 20.13)** | Reference  **1.50 (1.03, 2.18)**  **3.36 (2.34, 4.83)**  **8.59 (5.9, 12.53)**  **23.19 (14.93, 36.00)** |
| ***Men (vs. Women)*** | 0.99 (0.83, 1.19) | 0.96 (0.68, 1.37) | 1.00 (0.81, 1.23) |
| ***Ethnicity***  White  Indigenous  Other  Unknown | **1.77 (1.23, 2.55)**  **1.84 (1.22, 2.75)**  Reference  1.76 (0.94, 3.31) | 1.73 (0.83, 3.60)  **2.57 (1.17, 5.65)** Reference  2.86 (0.86, 9.54) | **1.79 (1.17, 2.72)**  1.60 (1.00, 2.57)  Reference  1.55 (0.75, 3.18) |
| ***Education level***  <Grade 10  Grade 10/11  Grade 12  Vocational /University  Unknown | 1.21 (0.89, 1.64)  **1.64 (1.27, 2.12)**  1.30 (1.00, 1.68)  Reference  1.32 (0.84, 2.05) | 1.02 (0.55, 1.90)  **1.83 (1.11, 3.02)**  1.22 (0.73, 2.05) Reference  0.79 (0.31, 2.05) | 1.28 (0.91, 1.81)  **1.59 (1.18, 2.13)**  1.33 (0.99, 1.79) Reference  1.47 (0.90, 2.41) |
| ***Year of methadone initiation***  1998 to 2000  2001 to 2005  2006 to 2010  2011 to 2015^[[1]](#footnote-1)^ | Reference  1.02 (0.85, 1.23)  0.83 (0.66, 1.05)  1.08 (0.79, 1.48) | Reference  0.98 (0.70, 1.36)  **0.33 (0.20, 0.54)**  **0.11 (0.03, 0.37)** | Reference  1.04 (0.84, 1.30)  1.17 (0.89, 1.53)  **1.91 (1.35, 2.71)** |
| ***Any offence in the year prior to enrolment***  None  1-2 offences  > 2 offences | Reference  1.07 (0.89, 1.28)  1.1 (0.89, 1.37) | Reference  1.25 (0.89, 1.76)  0.96 (0.62, 1.50) | Reference  1.01 (0.82, 1.25)  1.16 (0.9, 1.49) |
| ***# of offences after enrolment, per offence*** | 1.00 (0.99, 1.01) | 1.01 (0.99, 1.03) | 1.00 (0.99, 1.01) |
| ***Severe mental illness***  No Schizophrenia or Bipolar  Schizophrenia  Bipolar | Reference  **0.72 (0.57, 0.92)**  0.88 (0.72, 1.08) | Reference  0.88 (0.55, 1.40)  0.65 (0.41, 1.03) | Reference  0.68 (0.51, 0.89)  0.94 (0.75, 1.19) |
| ***MSP services (NSMD related) in the five-year period prior to enrolment***  Low^[[2]](#footnote-2)^ (≤ 2)  Medium (3 to 10)  High (≥11) | Reference  0.95 (0.79, 1.15)  0.89 (0.72, 1.12) | Reference  0.84 (0.59, 1.20)  **0.52 (0.32, 0.83)** | Reference  0.99 (0.79, 1.23)  1.05 (0.81, 1.35) |
| ***MSP services (SUD related) in the five-year period prior to enrolment***  Low^[[3]](#footnote-3)^ (≤ 4)  Medium (5 to 13)  High (≥14) | Reference  **1.31 (1.10, 1.57)**  1.00 (0.83, 1.21) | Reference  **1.52 (1.08, 2.13)**  0.99 (0.69, 1.41) | Reference  **1.25 (1.01, 1.54)**  1.01 (0.82, 1.26) |
| ***MSP services (non-psychiatric) in the five-year period prior to enrolment***  Low^[[4]](#footnote-4)^ (≤ 69)  Medium (70 to 139)  High (≥140) | Reference  **1.37 (1.12, 1.67)**  **2.05 (1.66, 2.52)** | Reference  **1.70 (1.17, 2.48)**  **2.30 (1.52, 3.49)** | Reference  **1.27 (1.01, 1.61)**  **1.97 (1.55, 2.50)** |

AHR: Adjusted Hazard Ratio; CI: Confidence Interval; MSP: Medical Services Plan; NSMD: Non-Substance Mental Disorder; SUD: Substance Use Disorder

1. -2015 included only three months (January to March) of data [↑](#footnote-ref-1)
2. -50^th^ & 75^th^ percentile was used to categorize into low, medium and high groups. [↑](#footnote-ref-2)
3. -50^th^ & 75^th^ percentile was used to categorize into low, medium and high groups [↑](#footnote-ref-3)
4. -50^th^ & 75^th^ percentile was used to categorize into low, medium and high groups [↑](#footnote-ref-4)
