# Supplementary material for: Methadone maintenance treatment and mortality in people with criminal convictions: A population-based retrospective cohort study from Canada
Source: PLoS Med. 2018 Jul 31;15(7):e1002625. doi: 10.1371/journal.pmed.1002625 (PMC6067717; doi:10.1371/journal.pmed.1002625)
Supplement: S4 Table — AHR, adjusted hazard ratio. (DOCX) [file pmed.1002625.s006.docx]

**S4 Table: AHR estimates of methadone and other predictors on external cause-specific mortality among 14,530 convicted offenders from BC, 1998–2015. AHR, adjusted hazard ratio.**

| **Variables** | **2: All external cause mortality (n=504)**  **AHR (95% CI)** | **2A: Accidental poisoning (n=355)**  **AHR (95% CI)** | **2B: Intentional self-harm (n=53)**  **AHR (95% CI)** | **2C: Other external causes (n=96)**  **AHR (95% CI)** |
| --- | --- | --- | --- | --- |
| ***Methadone (medicated period)*** | **0.41 (0.33, 0.51)** | **0.39 (0.30, 0.50)** | **0.36 (0.18, 0.70)** | **0.54 (0.34, 0.85)** |
| ***Age groups (years)***  18 < 25  25 < 35  35 < 45  45 < 55  ≥ 55 | Reference  1.19 (0.89, 1.58)  **1.55 (1.16, 2.06)**  **1.63 (1.14, 2.34)**  **3.07 (1.71, 5.51)** | Reference  1.38 (0.97, 1.98)  **1.77 (1.23, 2.53)**  **1.93 (1.24, 3.00)**  **3.04 (1.46, 6.33)** | Reference  0.72 (0.32, 1.60)  1.14 (0.54, 2.43)  0.89 (0.32, 2.51)  1.26 (0.17, 9.53) | Reference  0.98 (0.53, 1.81)  1.21 (0.65, 2.26)  1.32 (0.60, 2.93)  **4.56 (1.48, 14.03)** |
| ***Men (vs. Women)*** | **1.50 (1.20, 1.88)** | **1.44 (1.10, 1.88)** | 1.68 (0.81, 3.49) | 1.68 (1.00, 2.82) |
| ***Ethnicity***  White  Indigenous  Other  Unknown | 1.10 (0.78, 1.53)  1.05 (0.71, 1.57) Reference  1.34 (0.61, 2.95) | 1.09 (0.73, 1.62)  1.07 (0.66, 1.72)  Reference  1.69 (0.66, 4.33) | 1.27 (0.46, 3.52)  0.89 (0.24, 3.34) Reference  1.32 (0.19, 9.13) | 1.05 (0.5, 2.2)  1.08 (0.45, 2.57) Reference  0.54 (0.06, 4.64) |
| ***Education level***  <Grade 10  Grade 10/11  Grade 12  Vocational /University  Unknown | 1.02 (0.73, 1.44)  1.05 (0.79, 1.40)  1.04 (0.78, 1.39) Reference  **0.53 (0.28, 0.98)** | 0.98 (0.66, 1.45)  0.95 (0.68, 1.33)  0.96 (0.69, 1.34)  Reference  **0.34 (0.15, 0.77)** | 0.51 (0.17, 1.57)  0.86 (0.39, 1.90)  0.63 (0.27, 1.44) Reference  0.93 (0.26, 3.40) | 2.18 (0.83, 5.71)  2.13 (0.89, 5.12)  2.36 (0.99, 5.63) Reference  1.51 (0.36, 6.37) |
| ***Year of methadone initiation***  1998 to 2000  2001 to 2005  2006 to 2010  2011 to 2015^[[1]](#footnote-1)^ | Reference  0.87 (0.70, 1.08)  0.82 (0.64, 1.06)  **0.63 (0.43, 0.93)** | Reference  **0.76 (0.59, 0.98)**  0.75 (0.56, 1.01)  0.68 (0.43, 1.07) | Reference  0.98 (0.50, 1.92)  1.18 (0.55, 2.53)  0.98 (0.36, 2.69) | Reference  1.31 (0.80, 2.16)  0.99 (0.56, 1.75)  0.36 (0.13, 1.04) |
| ***Any offence in the year prior to enrolment***  None  1-2 offences  > 2 offences | Reference  1.02 (0.83, 1.27)  0.96 (0.74, 1.24) | Reference  0.99 (0.77, 1.28)  0.86 (0.62, 1.18) | Reference  1.47 (0.78, 2.79)  1.04 (0.44, 2.44) | Reference  0.92 (0.55, 1.54)  1.31 (0.79, 2.17) |
| ***# of offences after enrolment, per offence*** | 1.01 (1.00, 1.02) | 1.01 (1.00, 1.02) | 0.97 (0.92, 1.02) | 1.02 (1.00, 1.04) |
| ***Severe mental illness***  No Schizophrenia or Bipolar  Schizophrenia  Bipolar | Reference  1.16 (0.91, 1.47)  1.02 (0.80, 1.28) | Reference  1.19 (0.89, 1.60)  1.00 (0.76, 1.33) | Reference  **2.55 (1.33, 4.91)**  1.79 (0.89, 3.62) | Reference  0.60 (0.34, 1.08)  0.78 (0.45, 1.36) |
| ***MSP services (NSMD related) in the five-year period prior to enrolment***  Low^[[2]](#footnote-2)^ (≤ 2)  Medium (3 to 10)  High (≥11) | Reference  **1.44 (1.13, 1.84)**  **2.00 (1.54, 2.60)** | Reference  **1.41 (1.05, 1.88)**  **2.02 (1.47, 2.78)** | Reference  1.98 (0.93, 4.18)  1.91 (0.86, 4.24) | Reference  1.29 (0.76, 2.2)  **1.96 (1.11, 3.46)** |
| ***MSP services (SUD related) in the five-year period prior to enrolment***  Low^[[3]](#footnote-3)^ (≤ 4)  Medium (5 to 13)  High (≥14) | Reference  1.13 (0.90, 1.41)  1.07 (0.85, 1.35) | Reference  0.97 (0.73, 1.27)  0.99 (0.76, 1.29**)** | Reference  1.43 (0.73, 2.83)  1.19 (0.56, 2.50) | Reference  1.7 (1.03, 2.83)  1.39 (0.80, 2.43) |
| ***MSP services (non-psychiatric) in the five-year period prior to enrolment***  Low^[[4]](#footnote-4)^ (≤ 69)  Medium (70 to 139)  High (≥140) | Reference  1.07 (0.84, 1.37)  **1.37 (1.04, 1.80)** | Reference  1.06 (0.79, 1.42)  **1.48 (1.06, 2.06)** | Reference  0.94 (0.45, 1.96)  0.95 (0.42, 2.11) | Reference  1.21 (0.71, 2.04)  1.24 (0.67, 2.33) |

AHR: Adjusted Hazard Ratio; CI: Confidence Interval; MSP: Medical Services Plan; NSMD: Non-Substance Mental Disorder; SUD: Substance Use Disorder

1. -2015 included only three months (January to March) of data [↑](#footnote-ref-1)
2. -50^th^ & 75^th^ percentile was used to categorize into low, medium and high groups. [↑](#footnote-ref-2)
3. -50^th^ & 75^th^ percentile was used to categorize into low, medium and high groups [↑](#footnote-ref-3)
4. -50^th^ & 75^th^ percentile was used to categorize into low, medium and high groups [↑](#footnote-ref-4)
