# Supplementary material for: Methadone maintenance treatment and mortality in people with criminal convictions: A population-based retrospective cohort study from Canada
Source: PLoS Med. 2018 Jul 31;15(7):e1002625. doi: 10.1371/journal.pmed.1002625 (PMC6067717; doi:10.1371/journal.pmed.1002625)
Supplement: S5 Table — BC, British Columbia; HR, hazard ratio. (DOCX) [file pmed.1002625.s007.docx]

**S5 Table: Competing-risks regression analysis to estimate sub-HR of dispensed methadone (versus nondispensed methadone) on cause-specific mortality among 14,530 convicted offenders from BC, 1998–2015. BC, British Columbia; HR, hazard ratio.**

| **Type of death** | **Competing-risks regression^[[1]](#footnote-1)^** | **Cause-specific Cox Regression^[[2]](#footnote-2)^** |
| --- | --- | --- |
|  | **Unadjusted SHR (95% CI)** | **Unadjusted HR (95% CI)** |
| **1: Non-external causes (n=771)** | **0.32 (0.27, 0.39)** | **0.32 (0.26, 0.38)** |
| 1A: Infectious diseases (n=190) | **0.24 (0.16, 0.36)** | **0.23 (0.15, 0.35)** |
| 1B: Other non-external causes (n=581) | **0.35 (0.29, 0.44)** | **0.35 (0.28, 0.43)** |
|  |  |  |
| **2: External causes (n=504)** | **0.46 (0.38, 0.57)** | **0.45 (0.37, 0.55)** |
| 2A: Accidental poisoning (n=355) | **0.44 (0.34, 0.56)** | **0.43 (0.33, 0.55)** |
| 2B: Intentional self-harm (n=53) | **0.41 (0.21, 0.79)** | **0.40 (0.21, 0.77)** |
| 2C: Other external causes (n=96) | **0.59 (0.38, 0.92)** | **0.57 (0.37, 0.90)** |

CI: Confidence Interval; HR: Hazard Ratio; SHR: Sub Hazard Ratio

Note:

No violation of PH assumption for the variable methadone in all-cause model and cause–specific model

1. - Fine and Gray (1999) method was used. [↑](#footnote-ref-1)
2. -UHR from the Cox models were previously reported in Table 3 and repeated here for illustrative purposes only. [↑](#footnote-ref-2)
