# Supplementary material for: Methadone maintenance treatment and mortality in people with criminal convictions: A population-based retrospective cohort study from Canada
Source: PLoS Med. 2018 Jul 31;15(7):e1002625. doi: 10.1371/journal.pmed.1002625 (PMC6067717; doi:10.1371/journal.pmed.1002625)
Supplement: S6 Table — AHR, adjusted hazard ratio. (DOCX) [file pmed.1002625.s008.docx]

**S6 Table: AHR estimates of medicated methadone and other predictors on all-cause deaths among 7,722 convicted offenders from BC, 2007–2015. AHR, adjusted hazard ratio.**

| **Variables** | **All methadone recipients between 2007 and 2015 (n=7,722)^[[1]](#footnote-1)^**  **AHR (95% CI^[[2]](#footnote-2)^)** | **Methadone recipients between 2007 and 2015 with custody involvement (n=2,905)^[[3]](#footnote-3)^**  **AHR (95% CI)** |
| --- | --- | --- |
| ***Methadone (medicated period)*** | **0.31 (0.24, 0.40)** | **0.27 (0.15, 0.51)** |
| ***Age groups (years)***  18 < 25  25 < 35  35 < 45  45 < 55  ≥ 55 | Reference  1.05 (0.68, 1.64)  **1.82 (1.17, 2.82)**  **2.89 (1.81, 4.63)**  **8.99 (5.30, 15.25)** | Reference  1.55 (0.69, 3.49)  **2.53 (1.09, 5.84)**  **3.18 (1.16, 8.70)**  4.41 (0.89, 21.87) |
| ***Men (vs. Women)*** | 1.19 (0.89, 1.60) | 0.66 (0.39, 1.11) |
| ***Ethnicity***  White  Indigenous  Other  Unknown | 1.51 (0.91, 2.53)  1.63 (0.92, 2.89)  Reference  1.82 (0.85, 3.87) | 1.64 (0.57, 4.69)  1.73 (0.57, 5.26)  Reference  4.14 (0.40, 43.41) |
| ***Education level***  <Grade 10  Grade 10/11  Grade 12  Vocational /University  Unknown | 0.94 (0.59, 1.51)  1.29 (0.89, 1.87)  1.12 (0.78, 1.63)  Reference  0.93 (0.53, 1.65) | 1.36 (0.62, 2.96)  1.09 (0.54, 2.19)  0.81 (0.4, 1.66)  Reference  0.29 (0.03, 3.17) |
| ***Year of methadone initiation***  2007 to 2010  2011 to 2015^[[4]](#footnote-4)^ | Reference  0.91 (0.69, 1.18) | Reference  0.95 (0.48, 1.88) |
| ***Any offence in the year prior to enrolment***  None  1-2 offences  > 2 offences | Reference  0.86 (0.65, 1.15)  **0.63 (0.41, 0.98)** | Reference  0.98 (0.59, 1.62)  0.73 (0.40, 1.35) |
| ***# of offences after enrolment, per offence*** | 1.01 (0.95, 1.09) | 0.99 (0.91, 1.07) |
| ***# of custody admissions after enrolment, per admission*** | 1.02 (0.90, 1.16) | 1.12 (0.99, 1.27) |
| ***Severe mental illness***  No Schizophrenia or Bipolar  Schizophrenia  Bipolar | **Reference**  1.16 (0.86, 1.58)  1.25 (0.94, 1.67) | Reference  0.89 (0.48, 1.67)  1.41 (0.82, 2.41) |
| ***MSP services (NSMD related) in the five-year period prior to enrolment***  Low^[[5]](#footnote-5)^ (≤ 2)  Medium (3 to 10)  High (≥11) | Reference  **1.60 (1.20, 2.13)**  **1.41 (1.01, 1.97)** | Reference  **1.89 (1.11, 3.22)**  1.37 (0.69, 2.69) |
| ***MSP services (SUD related) in the five-year period prior to enrolment***  Low^[[6]](#footnote-6)^ (≤ 4)  Medium (5 to 13)  High (≥14) | Reference  **1.37 (1.03, 1.82)**  1.29 (0.96, 1.74) | Reference  1.17 (0.64, 2.13)  **1.89 (1.12, 3.18)** |
| ***MSP services (non-psychiatric) in the five-year period prior to enrolment***  Low^[[7]](#footnote-7)^ (≤ 69)  Medium (70 to 139)  High (≥140) | Reference  0.88 (0.65, 1.2)  1.11 (0.80, 1.53) | **Reference**  0.67 (0.37, 1.21)  0.65 (0.35, 1.21) |

AHR: Adjusted Hazard Ratio; CI: Confidence Interval; MSP: Medical Services Plan; NSMD: Non-Substance Mental Disorder; SUD: Substance Use Disorder

1. - Restricted to methadone recipients who initiated methadone between 2007 and 2015. This subgroup represented 7, 722 individuals who were accounted for 321 deaths (Infectious diseases: 23, Neoplasms: 54, Other non-external causes: 110, Accidental poisoning: 93, Intentional self-harm: 17 & Other external causes: 24). [↑](#footnote-ref-1)
2. -Robust estimator was used to calculate standard error and the confidence intervals for AHR estimates. [↑](#footnote-ref-2)
3. -Restricted to methadone recipients who initiated methadone between 2007 and 2015 and had at least one custody admission following methadone initiation. This subgroup represented 2,905 individuals who were accounted for 82 deaths (Infectious diseases: 7, Neoplasms: 5, Other non-external causes: 36, Accidental poisoning: 27, Intentional self-harm: 3 & Other external causes: 4). [↑](#footnote-ref-3)
4. -2015 included only three months (January to March) of data [↑](#footnote-ref-4)
5. -50^th^ & 75^th^ percentile was used to categorize into low, medium and high groups. [↑](#footnote-ref-5)
6. -50^th^ & 75^th^ percentile was used to categorize into low, medium and high groups [↑](#footnote-ref-6)
7. -50^th^ & 75^th^ percentile was used to categorize into low, medium and high groups [↑](#footnote-ref-7)
