# Supplementary material for: Methadone maintenance treatment and mortality in people with criminal convictions: A population-based retrospective cohort study from Canada
Source: PLoS Med. 2018 Jul 31;15(7):e1002625. doi: 10.1371/journal.pmed.1002625 (PMC6067717; doi:10.1371/journal.pmed.1002625)
Supplement: S7 Table — AHR, adjusted hazard ratio; BC, British Columbia. (DOCX) [file pmed.1002625.s009.docx]

**S7 Table: AHR estimates of medicated methadone and other predictors on HIV-related deaths among 14,530 convicted offenders from BC, 1998–2015. AHR, adjusted hazard ratio; BC, British Columbia.**

| **Variables** | **All methadone recipients between 2007 and 2015 (n=114)^[[1]](#footnote-1)^**  **AHR (95% CI^[[2]](#footnote-2)^)** |
| --- | --- |
| ***Methadone (medicated period)*** | **0.19 (0.11, 0.33)** |
| ***Age groups (years)***  18 < 25  25 < 35  35 < 45  45 < 55  ≥ 55 | Reference  **2.33 (1.14, 4.77)**  **4.49 (2.28, 8.84)**  **6.67 (3.16, 14.09)**  2.34 (0.29, 18.83) |
| ***Men (vs. Women)*** | 0.93 (0.59, 1.46) |
| ***Ethnicity***  White  Indigenous  Other  Unknown | 1.43 (0.60, 3.41)  **2.66 (1.05, 6.78)**  Reference  0.84 (0.09, 7.52) |
| ***Education level***  <Grade 10  Grade 10/11  Grade 12  Vocational /University  Unknown | 1.80 (0.76, 4.24)  **2.29 (1.07, 4.88)**  1.82 (0.85, 3.92)  Reference  1.48 (0.36, 6.02) |
| ***Year of methadone initiation***  1998 to 2000  2001 to 2005  2006 to 2010  2011 to 2015^[[3]](#footnote-3)^ | Reference  0.82 (0.54, 1.23)  **0.19 (0.10, 0.37)**  **0.09 (0.02, 0.35)** |
| ***Any offence in the year prior to enrolment***  None  1-2 offences  > 2 offences | Reference  1.31 (0.86, 2.02)  0.90 (0.51, 1.57) |
| ***# of offences after enrolment, per offence*** | 1.02 (1.00, 1.04) |
| ***Severe mental illness***  No Schizophrenia or Bipolar  Schizophrenia  Bipolar | Reference  0.65 (0.35, 1.23)  0.56 (0.30, 1.03) |
| ***MSP services (NSMD related) in the five-year period prior to enrolment***  Low^[[4]](#footnote-4)^ (≤ 2)  Medium (3 to 10)  High (≥11) | Reference  0.65 (0.41, 1.03)  **0.37 (0.20, 0.70)** |
| ***MSP services (SUD related) in the five-year period prior to enrolment***  Low^[[5]](#footnote-5)^ (≤ 4)  Medium (5 to 13)  High (≥14) | Reference  **1.70 (1.10, 2.62)**  1.01 (0.64, 1.60) |
| ***MSP services (non-psychiatric) in the five-year period prior to enrolment***  Low^[[6]](#footnote-6)^ (≤ 69)  Medium (70 to 139)  High (≥140) | Reference  **2.06 (1.28, 3.31)**  **2.82 (1.61, 4.93)** |

AHR: Adjusted Hazard Ratio; CI: Confidence Interval; MSP: Medical Services Plan; NSMD: Non-Substance Mental Disorder; SUD: Substance Use Disorder

1. - Restricted to HIV-related deaths (among 190 infectious and parasitic disease-related deaths, 114 deaths were attributed to HIV, 62 were related to viral hepatitis and the rest 14 were related to parasitic diseases). [↑](#footnote-ref-1)
2. -Robust estimator was used to calculate standard error and the confidence intervals for AHR estimates. [↑](#footnote-ref-2)
3. -2015 included only three months (January to March) of data [↑](#footnote-ref-3)
4. -50^th^ & 75^th^ percentile was used to categorize into low, medium and high groups. [↑](#footnote-ref-4)
5. -50^th^ & 75^th^ percentile was used to categorize into low, medium and high groups [↑](#footnote-ref-5)
6. -50^th^ & 75^th^ percentile was used to categorize into low, medium and high groups [↑](#footnote-ref-6)
