# Supplementary material for: Methadone maintenance treatment and mortality in people with criminal convictions: A population-based retrospective cohort study from Canada
Source: PLoS Med. 2018 Jul 31;15(7):e1002625. doi: 10.1371/journal.pmed.1002625 (PMC6067717; doi:10.1371/journal.pmed.1002625)
Supplement: S8 Table — (DOCX) [file pmed.1002625.s010.docx]

**S8 Table: Sensitivity analysis to estimate the effect of methadone on mortality with inflated time on last methadone fill among 14,530 convicted offenders from BC, 1998–2015.**

| **Cause of Death** | **MMT inflated time (days)** | **UHR (95% CI^[[1]](#footnote-1)^)** | **AHR^[[2]](#footnote-2)^ (95% CI)** |
| --- | --- | --- | --- |
| All-cause mortality (n=1,275^[[3]](#footnote-3)^) | 3-days inflated time on last MMT | 0.51 (0.45, 0.58) | 0.45 (0.40, 0.52) |
|  | 7-days inflated time on last MMT | 0.61 (0.54, 0.69) | 0.55 (0.48, 0.62) |
| Non-external causes mortality (n=771) | 3-days inflated time on last MMT | 0.46 (0.39, 0.55) | 0.41 (0.34, 0.48) |
|  | 7-days inflated time on last MMT | 0.60 (0.51, 0.70) | 0.52 (0.45, 0.52) |
| External causes mortality (n=504) | 3-days inflated time on last MMT | 0.59 (0.48, 0.71) | 0.54 (0.44, 0.66) |
|  | 7-days inflated time on last MMT | 0.64 (0.53, 0.77) | 0.59 (0.48, 0.71) |

AHR: Adjusted Hazard Ratio; CI: Confidence Interval; UHR: Undusted Hazard Ratio

1. -Robust estimator was used to calculate standard error and the confidence intervals for both UHR and AHR estimates. [↑](#footnote-ref-1)
2. -Separate multivariable cox regression (all-cause mortality) was conducted for 3-days and 7 days inflated time on last MMT refill. Each multivariable model was controlled for: age (18 < 25 years, 25 < 35 years, 35 < 45 years, 45 < 55 years, and ≥55), gender (men & women), ethnicity (White, Indigenous & Other), education (<Grade 10, Grade 10/11, Grade 12 & Vocational /University), initiation period (1998 to 2000, 2001 to 2005, 2006 to 2010 & 2011 to 2015), prior offences (None, 1-2 offences & > 2 offences), severe mental illness (No Schizophrenia or Bipolar, Schizophrenia & Bipolar), prior NSMD related services (low, medium and high), prior SUD related services (low, medium and high), and prior non-psychiatric services (low, medium and high). [↑](#footnote-ref-2)
3. -Values in parenthesis represents number of deaths, not number of individuals. [↑](#footnote-ref-3)
